# Isorhamnetin: Reviewing Recent Developments in Anticancer Mechanisms and Nanoformulation-Driven Delivery

**DOI:** 10.3390/ijms26157381

**Published:** 2025-07-30

**Authors:** Juie Nahushkumar Rana, Kainat Gul, Sohail Mumtaz

**Affiliations:** 1 Fels Cancer Institute for Personalized Medicine, Lewis Katz School of Medicine at Temple University, Philadelphia, PA 19140, USA; 2 Department of Botany, Hazara University, Mansehra 21120, Pakistan; 3 Department of Chemical and Biological Engineering, Gachon University, 1342 Seongnamdaero, Sujeong-gu, Seongnam-si 13120, Republic of Korea

**Keywords:** isorhamnetin flavonoids, anticancer, nanoformulation, apoptosis, metastasis

## Abstract

Natural compounds, particularly flavonoids, have emerged as promising anticancer agents due to their various biological activities and no or negligible toxicity towards healthy tissues. Among these, isorhamnetin, a methylated flavonoid, has gained significant attention for its potential to target multiple cancer hallmarks. This review comprehensively explores the mechanisms by which isorhamnetin exerts its anticancer effects, including cell cycle regulation, apoptosis, suppression of metastasis and angiogenesis, and modulation of oxidative stress and inflammation. Notably, isorhamnetin arrests cancer cell proliferation by regulating cyclins, and CDKs induce apoptosis via caspase activation and mitochondrial dysfunction. It inhibits metastatic progression by downregulating MMPs, VEGF, and epithelial–mesenchymal transition (EMT) markers. Furthermore, its antioxidant and anti-inflammatory properties mitigate reactive oxygen species (ROS) and pro-inflammatory cytokines, restricting cancer progression and modulating tumor microenvironments. Combining isorhamnetin with other treatments was also discussed to overcome multidrug resistance. Importantly, this review integrates the recent literature (2022–2024) and highlights isorhamnetin’s roles in modulating cancer-specific signaling pathways, immune evasion, tumor microenvironment dynamics, and combination therapies. We also discuss nanoformulation-based strategies that significantly enhance isorhamnetin’s delivery and bioavailability. This positions isorhamnetin as a promising adjunct in modern oncology, capable of improving therapeutic outcomes when used alone or in synergy with conventional treatments. The future perspectives and potential research directions were also summarized. By consolidating current knowledge and identifying critical research gaps, this review positions Isorhamnetin as a potent and versatile candidate in modern oncology, offering a pathway toward safer and more effective cancer treatment strategies.

## 1. Introduction

Cancer remains one of the leading causes of morbidity and mortality worldwide, imposing a significant burden on global health systems and societies [1]. There were nearly 20 million new cancer cases and 9.7 million cancer deaths worldwide. Global cancer statistics for 2022 reveal substantial regional disparities in incidence and mortality rates by cancer, with projections indicating an increasing global problem by 2050 [2]. These results underline the necessity for region-specific approaches to address the rising cancer burden. Furthermore, the growing incidence and mortality rates highlight the importance of progressing research into innovative treatment opportunities to alleviate the future impact of cancer. This alarming trend emphasizes the pressing need to develop new and effective treatment strategies [3]. Advancing research and information to explore innovative and less toxic therapeutic approaches is crucial for improving cancer patients’ global survival outcomes and quality of life [4].

The search for effective and less toxic cancer treatments has directed attention toward natural compounds derived from plants, fungi, and marine organisms. These bioactive molecules, often secondary metabolites, exhibit various pharmacological activities, including antioxidant, anti-inflammatory, and antitumor properties [5,6]. Among these, flavonoids, alkaloids, terpenoids, and phenolics have garnered significant interest due to their ability to modulate cancer-related pathways such as cell cycle regulation, apoptosis, angiogenesis inhibition, and immune system enhancement [7]. Natural compounds offer distinct advantages in cancer therapy, including a broad spectrum of activity and the potential to reduce side effects associated with conventional chemotherapy [8]. Moreover, they often exhibit synergistic effects when combined with existing treatments. Despite their promise, challenges such as limited bioavailability, variability in potency, and the need for large-scale clinical validation persist. However, advances in biotechnology and nanotechnology are paving the way for harnessing the full potential of natural compounds, making them integral to the future of cancer research and personalized medicine [8,9].

Over recent years, the utilization of plant-derived compounds in therapeutic applications has increased substantially [10,11,12,13]. This is because of their efficacy and reduced side effects compared to synthetic drugs [14,15,16]. Among these, flavonoids have garnered significant attention. Approximately 4000 distinct flavonoids have been identified, many of which exhibit potent medicinal properties, including antioxidant, antiviral, anti-inflammatory, and anticancer activities [10,17]. Their biological versatility highlights their potential as valuable agents in developing novel disease treatments. A particularly promising compound in this regard is isorhamnetin, a flavonoid that has emerged as a potent anticancer agent.

Despite a growing body of literature on isorhamnetin, recent advances in its molecular mechanisms, combination strategies, and therapeutic delivery approaches remain fragmented and under-integrated [9]. Several existing reviews have summarized its general pharmacological effects; however, they often lack depth in cancer-type-specific mechanisms, immune modulation, and translational potential. This review is motivated by the need to provide a comprehensive and mechanistically stratified synthesis of isorhamnetin’s anticancer activity, integrating the most recent discoveries up to mid-2025. In particular, we emphasize its role in modulating apoptosis, reversing EMT, enhancing chemoradiotherapy, and its behavior in glycosidic forms, as well as the implications of these findings in various cancer models. By critically analyzing scientific literature and organizing the findings into a structured, accessible format, this review aims to offer a next-generation reference for both researchers and clinicians exploring isorhamnetin’s therapeutic promise.

### Methods

The literature review was conducted using a structured approach across multiple databases, including PubMed, Scopus, Web of Science, and Google Scholar, covering publications up to June 2025. The following keywords and Boolean operators were used: “isorhamnetin” AND (“anticancer” OR “apoptosis” OR “metastasis” OR “nanoformulation” OR “drug delivery” OR “immune modulation”). Only peer-reviewed English-language publications were considered. We included original research articles, recent high-impact reviews, and preclinical and clinical studies involving isorhamnetin or its glycosidic forms, either as monotherapy or in combination with other agents. Studies focusing solely on quercetin or other flavonols were excluded unless they provided comparative insights on isorhamnetin. Reference mining from key papers was also used to identify additional relevant sources. Duplicates and non-scientific sources were removed. A total of over 400 peer-reviewed publications were critically evaluated and integrated into this review based on their scientific merit and relevance.

## 2. Isorhamnetin

Isorhamnetin, a naturally occurring flavonoid, is predominantly found in various plant species of leaves, flowers, and fruits [18]. Its ability to modulate key signaling pathways and regulate immune response makes it an exciting candidate for further examination as a therapeutic option in cancer treatment [19,20]. Flavonoids, especially isorhamnetin, were identified as key contributors to anticancer activity [21].

### 2.1. Chemical Structure of Isorhamnetin and Its Significance in Biomedical Applications

**Figure 1:**
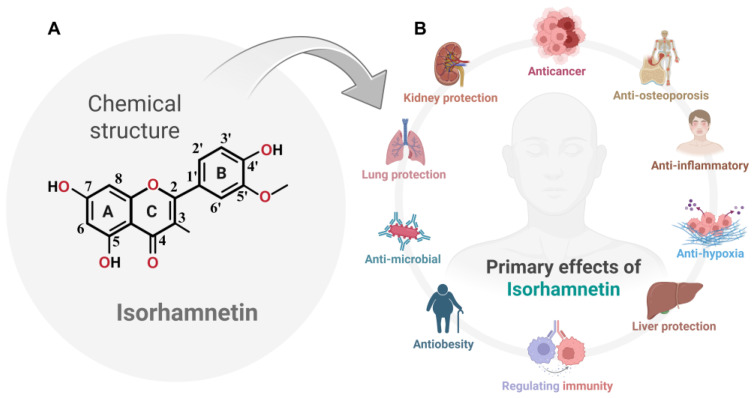
(**A**) The chemical structure of isorhamnetin, a naturally occurring flavonol, is depicted with standard IUPAC carbon numbering and labeled rings (A, B, and C) and key functional groups relevant to its bioactivity. (**B**) The major pharmacological effects of isorhamnetin are illustrated, highlighting its anticancer, anti-inflammatory, and anti-osteoporotic activities. Isorhamnetin also exerts protective roles in liver, kidney, and lung function, mitigates hypoxic stress, and demonstrates antimicrobial, anti-obesity, and immunomodulatory properties. Together, these features underscore its potential as a multifunctional therapeutic agent. The figure was prepared using BioRender.

Isorhamnetin, per PubChem identifier CID 5281654, is chemically described as *3,5,7-trihydroxy-2-(4-hydroxy-3-methoxyphenyl)-4H-chromen-4-one*, with the molecular formula C_16_H_12_O_7_ and molecular weight of 316.26 g·mol^−1^. It is a *3′-O*-methylated flavonol, derived from quercetin via replacement of the 3′-OH with -OCH_3_ [22]. Isorhamnetin, a naturally occurring *3′-O*-methylated flavonol, belongs to the flavonoid family and is categorized by a C_15_ skeleton encompassing two aromatic rings connected by a heterocyclic pyran ring, as shown in Figure 1A. These structural features of isorhamnetin confer important biological implications, allowing isorhamnetin to act as an effective antioxidant and anti-inflammatory molecule. Its ability to scavenge reactive oxygen species (ROS) and influence cellular signaling pathways makes it an effective candidate for various biological applications, particularly in cancer treatments [18]. Studies have demonstrated that isorhamnetin can inhibit tumor cell proliferation, induce apoptosis, and suppress metastasis by targeting key molecular pathways, such as PI3K/Akt, MAPK, and NF-κB. These anticancer properties and their natural abundance in plants like *Hippophae rhamnoides* L. and *Ginkgo biloba* L. highlight isorhamnetin’s potential as a lead compound for developing novel, plant-based chemotherapeutic agents [18].

In natural plant matrices, isorhamnetin predominantly exists in glycosylated forms, not as the free aglycone [23,24]. This distinction is critical, as the glycosides exhibit distinct solubility, absorption, and metabolic profiles compared to the aglycone. Common glycosides include isorhamnetin-3-O-rutinoside (also known as narcissin), which is found in *Hippophae rhamnoides* (sea buckthorn) and *Calendula officinalis* [25]; isorhamnetin-3-O-glucoside, identified in the leaves of *Ginkgo biloba* [18]; and isorhamnetin-3-O-robinobioside, which occurs in onions and other Allium species. Other notable derivatives include isorhamnetin-3-O-galactoside, reported in marigold (*Tagetes erecta*). These glycosidic forms are the biologically relevant constituents typically absorbed after ingestion and may be hydrolyzed in the gut or further metabolized in the liver. Consequently, any in vivo pharmacological activities of isorhamnetin are likely influenced by these glycosides or their metabolites rather than the aglycone alone [26].

Isorhamnetin (bioactive compound) has been widely recognized for its therapeutic potential, including antioxidant [27], Anticancer [28], anti-osteoporosis [29], anti-inflammatory [30], anti-hypoxia [18,31], liver protection [32], and cardioprotective properties [33], immunomodulation [34], anti-obesity [35], antimicrobial [36], lung protection [37], and kidney protection [38], making it an interesting candidate for numerous pharmacological applications. Its occurrence in diverse plant species underlines its ecological and biological significance (Figure 1B) and its potential for broader utilization in drug development. Although prior reviews have summarized the anticancer properties of isorhamnetin, our manuscript uniquely explores its multifaceted role in cancer biology, including recently characterized mechanisms of synergistic effects in combination treatments with chemotherapeutics and biologics [18,20,26,35,39]. We also emphasize novel delivery platforms, such as nanoformulations, that enhance pharmacokinetics and target specificity. By linking isorhamnetin’s molecular effects to both monotherapy and combinatorial contexts, we offer a forward-looking perspective aligned with clinical translational needs.

### 2.2. Isorhamnetin: Sources and Its Nutritional Significance

Isorhamnetin is a flavonoid found in various fruits, vegetables, and medicinal plants used daily and has received recognition for its significant health benefits. As a dietary component, it plays a vital role in facilitating key biological activities. Isorhamnetin is a flavonoid that is of significant use in the biomedical field, as shown in Figure 1B [40]. Isorhamnetin is widely distributed across numerous plant species, particularly in members of the *Asteraceae* and *Rosaceae* families [41], where it is often present at notably higher concentrations than in previously highlighted sources such as sea buckthorn or ginkgo [18]. Plants in these families—such as various herbs, leafy vegetables, and flowering plants—serve as rich natural sources. The phytochemical surveys demonstrate that many members of the *Asteraceae* family—such as *Calendula officinalis*—contain isorhamnetin glycosides at concentrations as high as 36.7 mg/g in florets [42]. In one Brazilian study, isorhamnetin derivatives in *Asteraceae* were observed at levels 6.8 to 16.2 times higher than in comparable species [41]. Additionally, Rubus and Fragaria berries (*Rosaceae*) serve as significant dietary sources, with USDA data confirming their flavonol content [43]. In contrast, sea buckthorn berries, while high in isorhamnetin rutinoside (~96–228 mg/100 g DW), represent only one of many rich sources [40].

Additionally, commonly consumed fruits such as apples, pears, grapes, and onions contribute to dietary intake of isorhamnetin, particularly through their skins, where flavonols tend to accumulate [43,44]. While these fruits serve as accessible nutritional sources, higher concentrations of isorhamnetin are found in various plants belonging to the *Asteraceae* and *Rosaceae* families, including *Calendula officinalis*, *Tagetes erecta*, and berries from the *Rubus* and *Fragaria genera* [42,45,46]. In particular, *Calendula officinalis* (Asteraceae) has been reported to contain up to 36.7 mg/g of isorhamnetin derivatives in its florets [42], far exceeding levels typically observed in sea buckthorn [47]. Therefore, current phytochemical surveys suggest that *Asteraceae* and *Rosaceae* plants may represent richer natural sources than previously emphasized medicinal plants like *Hippophae rhamnoides* or *Ginkgo biloba* [18]. The bioactive compound enriched nutritional component, including isorhamnetin, contributes to its therapeutic properties, including immune system support and anti-inflammatory effects [19]. Fruits like apples and pears, often consumed raw, are significant suppliers of isorhamnetin compounds in our daily diet [48,49]. It is primarily concentrated in the skin of these fruits, suggesting the importance of consuming them unpeeled to achieve the supreme health benefits [49]. Likewise, vegetables like onions and spinach are good sources of isorhamnetin [50,51]. In the medicinal plant realm, *Ginkgo biloba*, *Hippophae rhamnoides* L, and *Echinacea purpurea* stand out as essential sources of antioxidative properties and immune-boosting effects [28,52,53].

The existence of isorhamnetin compounds in various medicinal plants emphasizes its role in facilitating health, particularly acting as an antioxidant, which neutralizes excessive free radical formation and reduces oxidative stress, which is one of the key factors in aging and disease development [54,55]. Moreover, ongoing research suggests that isorhamnetin may exert protective effects on cardiovascular health, metabolic syndromes, and various types of cancer [39]. Consuming a diet rich in flavonoid-containing fruits, vegetables, and herbs supports general health and provides the body with compounds like isorhamnetin, which offers substantial health benefits against serious diseases (Figure 1B).

## 3. Mechanism of Action of Isorhamnetin in Cancers

Isorhamnetin, a bioactive flavonoid, has been shown to exert significant anticancer effects through multiple mechanisms. Biswas et al. [20] discussed mechanistic effects and delivery platforms in detail. While Biswas et al. [20] offered a foundational summary of isorhamnetin’s anticancer mechanisms and formulation efforts, our review builds upon and extends this work by integrating recent mechanistic findings and introducing isorhamnetin’s potential role in combination regimens and immune-targeted therapies.

Although several reviews have previously summarized the general pharmacological effects of isorhamnetin, this review provides a more mechanistic, integrative, and application-oriented synthesis [18,20,26,39]. Specifically, we highlight recent developments in combination therapies, illustrating how isorhamnetin enhances the efficacy of standard anticancer agents and contributes to overcoming multidrug resistance. Moreover, this review places special emphasis on immune-modulatory mechanisms, detailing the influence of isorhamnetin on NK cell activation, cytokine signaling, and checkpoint regulation. We also categorize cancer-type-specific pathways, mapping isorhamnetin’s effects on PI3K/AKT, MAPK, p53, EMT, and others across distinct tumor models. Finally, we explore recent advances in nanoformulation-based delivery strategies, addressing pharmacokinetic limitations and expanding the compound’s clinical potential. Together, these dimensions differentiate this work from existing reviews and offer a forward-looking perspective on isorhamnetin’s role in modern oncology. Its anticancer properties are mainly attributed to its ability to regulate cell cycle progression, induce apoptosis, inhibit angiogenesis, suppress metastasis, reduce oxidative stress, and modulate the tumor microenvironment. These mechanisms, discussed below, outline how isorhamnetin inhibits cancer cell growth and progression.

### 3.1. Effect of Isorhamnetin on Cell Cycle Regulations

**Figure 2:**
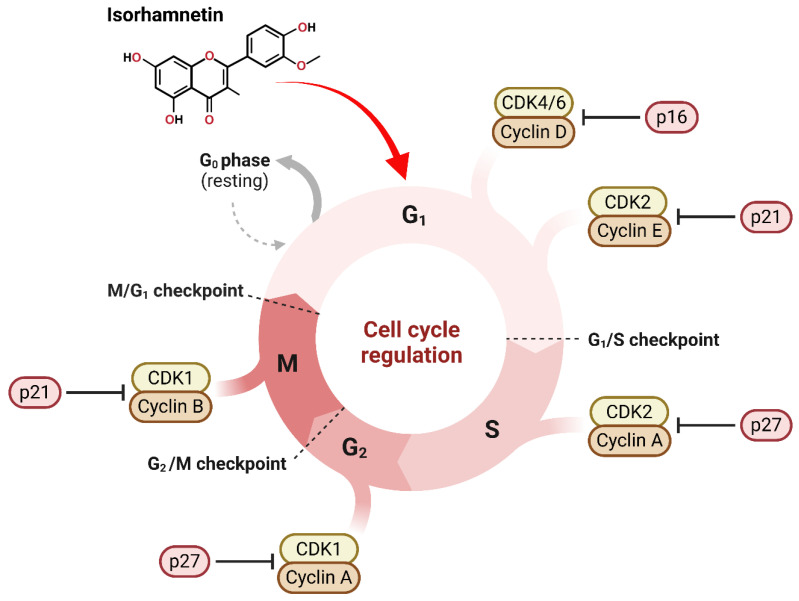
Isorhamnetin influences cell cycle regulation by interacting with various CDKs and cyclins in the cell. The cell cycle phases, including G_0_, which is also known as resting, G_1_, S, G_2_, and M phases, indicate important checkpoints that confirm proper cell development. Isorhamnetin can be useful in modulating CDKs and cyclins at different cycle phases. Isorhamnetin can inhibit CDK4/6-Cyclin D action by upregulating the p21 marker at the G_1_ phase. At the G_1_/S checkpoint, isorhamnetin suppresses CDK2-Cyclin E by upregulating the expression of p21. Similarly, in the S phase, CDK2-Cyclin A activity is inhibited via p27. Through the G_2_/M checkpoint, isorhamnetin reduces CDK1-Cyclin A activity by p27 expression, and at the M phase, the activity of CDK1-Cyclin B is inhibited by p21. This determines the potential of isorhamnetin, a bioactive compound, to influence cell cycle progression, possibly induce cell cycle arrest, and contribute to treating various cancers [26,56,62,63]. The figure was prepared using Biorender.

One of the primary mechanisms through which the isorhamnetin compound employs its anticancer effects is by influencing the cell cycle [56,57], as shown in Figure 2. The cell cycle is a controlled process that includes numerous checkpoints (G1/S checkpoint, G2/M checkpoint, M/G1 checkpoint) and regulatory proteins that confirm appropriate cell division [58,59]. Cell cycle dysregulation can lead to uncontrolled cell growth and eventually induce cancers [60]. On the other hand, the arrest of the cell cycle can be used to inhibit cancers [61]. Isorhamnetin can effectively affect cell cycle regulation through its interaction with several cyclin-dependent kinases (CDKs) and cyclins, as shown in Figure 2.

#### 3.1.1. Impact of Isorhamnetin on Cyclins and CDKs

Isorhamnetin has been shown to impact important cell cycle regulators involving cyclins and CDKs [28,64]. Cyclins are key proteins that control CDKs, which phosphorylate target proteins to progress the cell cycle [28]. Generally, the overexpression of cyclins and CDKs often results in unchecked cell division in cancers [65]. Cancer is characterized by dysregulated cell cycle control, allowing unchecked proliferation due to the evasion of inhibitory signals and reduced dependency on extrinsic growth factors. This leads to inappropriate cell division and tumor formation, often bypassing standard DNA damage response mechanisms and cell cycle checkpoints [65]. Isorhamnetin modulates the expression levels of numerous cyclins, mainly cyclin D1 and cyclin E, and their corresponding CDKs, such as CDK4 and CDK2 (Figure 2). By inhibiting these cyclins and CDKs, isorhamnetin successfully induces cell cycle arrests at critical checkpoints, leading to the inhibition of cancer cells or induction of apoptosis. A recent study by Yang et al. reported that isorhamnetin significantly affected the expression of cyclins and CDKs, mainly inducing G2/M phase cell cycle arrest in doxorubicin-resistant breast cancer cells by downregulating the Cyclin B1/CDK1 complex [62]. Additionally, isorhamnetin triggered increased ROS production and DNA damage, eventually disrupting cell proliferation and promoting apoptosis via regulation of AMPK/mTOR signaling pathways [62]. It is also reported that isorhamnetin can exhibit anticancer effects in human bladder cancer cells by inducing G2/M phase cell cycle arrest and apoptosis [56]. The isorhamnetin treatment effectively downregulated the cyclin B1 and Wee1 expressions while upregulating the CDK inhibitor p21, which leads to promoting G2/M arrest, eventually inducing apoptosis through ROS-dependent mitochondrial dysfunction, caspase activation, and AMPK signaling pathway activation [56].

#### 3.1.2. Arrest at G1/S and G2/M Phases

The G1/S and G2/M phases are critical checkpoints in the cell cycle, where the cell mainly decides whether to progress with DNA replication or enter mitosis. Pieces of evidence showed that isorhamnetin has been shown to induce cell cycle arrest at both these points [20,39,56,62,64,66]. In the G1/S phase, isorhamnetin inhibits the activity of cyclin D-CDK4 complexes, promoting activation of the retinoblastoma protein (pRb), which prevents the cell from progressing into the S phase [67,68]. In the G2/M phase, isorhamnetin affects the activity of cyclin B-CDK1 complexes, preventing the cells from entering mitosis [56]. This dual arrest at the G1/S and G2/M phases by isorhamnetin results in the reduction or inhibition of tumor growth. The study by Chen and Coworkers showed that isorhamnetin successfully induces cell cycle arrest at the G2/M phase in oral squamous cell carcinoma (OSCC) cells by downregulating cyclin B1 and CDC2, thus inhibiting cell proliferation and migration [66].

### 3.2. Apoptosis Induction Pathways

Apoptosis is programmed cell death, a natural mechanism by which the body eradicates damaged/abnormal cells [69]. Dysregulation of the apoptosis mechanism contributes to cancer cell survival and resistance to various treatments [70,71,72]. In the last few years, isorhamnetin has been shown to cause apoptosis in numerous cancer cell lines by triggering both intrinsic and extrinsic apoptotic pathways.

#### Activation of Intrinsic and Extrinsic Pathways by Isorhamnetin

**Figure 3:**
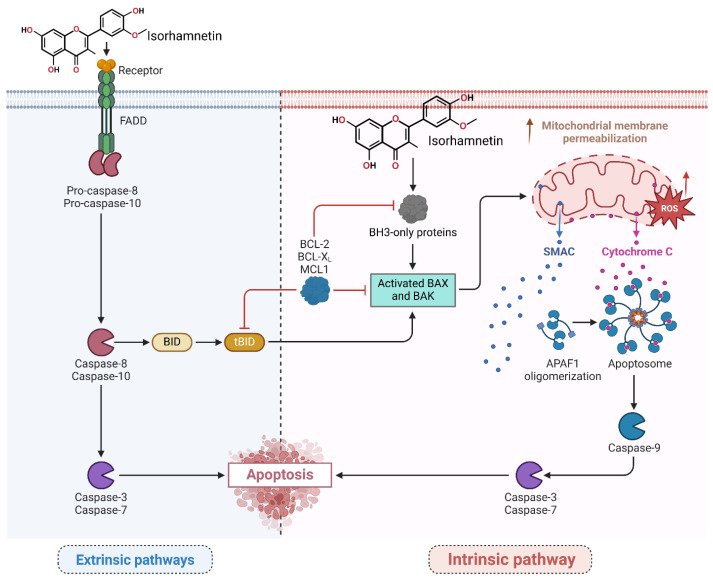
The isorhamnetin compound induces apoptosis in numerous tumor cells by triggering intrinsic and extrinsic-mediated pathways. In the extrinsic pathway, the isorhamnetin compound triggers the FAS receptor, activates FADD, and cleavages of pro-caspase-8/10. Stimulated caspase-8 then processes BID into tBID, which links the extrinsic pathway to the intrinsic pathway by increasing the permeabilization of the outer membrane of mitochondria. In the intrinsic pathway, isorhamnetin induced mitochondrial dysfunction by increasing endogenous ROS levels and disrupting the balance (upregulating) between pro-apoptotic (BAX, BAK) and (downregulating) anti-apoptotic (BCL-XL, BCL-2, MCL1) proteins. This scenario leads to cytochrome c and SMAC release from mitochondria. Cytochrome c forms the apoptosome with the APAF1 marker, leading to the activation of caspase-9. Both pathways meet to activate effector caspases (caspase-3 and caspase-7) and induce apoptosis by isorhamnetin [20,83,85,86]. The figure was prepared using Biorender.

The intrinsic apoptosis pathway can be activated through cellular stresses, such as DNA damage or oxidative stress, which releases pro-apoptotic proteins from the mitochondria [73,74,75,76]. Isorhamnetin triggers the intrinsic pathway by enhancing mitochondrial membrane permeability, eventually releasing cytochrome c, and the initiated caspase cascades [77], as shown in Figure 3. A recent study showed that isorhamnetin impacts intrinsic and extrinsic apoptotic pathways to effectively regulate cancer cell death [78]. In the intrinsic pathway, it upregulates the expression of BAX and BAK genes and downregulates BCL-2, BCL-XL, and MCL1 expression. Isorhamnetin (10 µM) increases the polar body extrusion rate of oocytes. Isorhamnetin treatment alleviates oxidative stress by reducing ROS levels and triggering SOD2 protein expression. Isorhamnetin facilitates oocyte maturation by alleviating oxidative stress, mitochondrial dysfunction, apoptosis, and endoplasmic reticulum (ER) stress [78,79], eventually improving oocyte quality and female infertility treatment [78,79].

Utilizing isorhamnetin in vitro model noticeably inhibited cell necrosis in severe acute pancreatitis, primarily by inhibiting mitochondrial ROS generation, preserving ATP production, preventing oxidative damage, and releasing mitochondrial DNA [80]. On the other hand, the extrinsic pathway is initiated by death receptor activation on the cell surface by isorhamnetin [81]. The isorhamnetin has been shown to upregulate the expression of death receptors, such as Fas and TRAIL (TNF-related apoptosis-inducing ligand) [80,82], thereby activating the extrinsic apoptotic pathway (Figure 3). Another study by Sun et al. reported that isorhamnetin attenuates apoptosis and promotes mitophagy, highlighting its potential therapeutic role in managing oxidative stress-induced apoptosis and related diseases [83,84]. Caspases are a family of cysteine proteases that play vital roles in executing apoptosis.

Isorhamnetin induces the activation of caspases, including caspase-3/8/9, which leads to the cleavage of key cellular pathways and the induction of cell apoptosis/death. This cascade modulation by isorhamnetin ultimately leads to cell death and the inhibition of tumor progression [87]. The study by Chen et al., by modulating endogenous ROS levels and enhancing oxidative stress responses, isorhamnetin interferes with the intrinsic apoptosis pathway, contributing to mitochondrial membrane stabilization and suppression of apoptotic signaling cascades [88]. The study’s findings indicate isorhamnetin’s potential as a therapeutic agent targeting dysregulated cell cycle and apoptotic pathways, particularly in resistant or aggressive cancer phenotypes such as OSCC [66]. Moreover, isorhamnetin treatment reveals anti-platelet aggregation and anti-thrombotic effects by decreasing ATP levels and mitochondrial dysfunction in platelets without affecting endogenous ROS levels [87]. A recent study showed that isorhamnetin effectively alleviates ferroptosis-mediated colitis by activating the NRF2/HO-1 pathway and chelating iron. It reduces oxidative stress, lipid peroxidation, and pro-inflammatory cytokines while enhancing glutathione levels and suppressing ferroptosis markers like ACSL4 and PTGS2. In vivo, isorhamnetin decreases inflammation, colon shortening, and disease activity in Dextran Sulfate Sodium (DSS)-induced colitis models, showcasing its potential as a therapeutic agent for ferroptosis-related colitis [89]. Isorhamnetin is effective in activating Nrf2, leading to the induction of antioxidant genes, and offers protection against oxidative stress in hepatocytes. This protective effect is mediated through Nrf2 activation and the enhancement of cellular antioxidant responses, making isorhamnetin a potential candidate for mitigating liver oxidative injury [90].

### 3.3. Suppression of Angiogenesis and Metastasis

**Figure 4:**
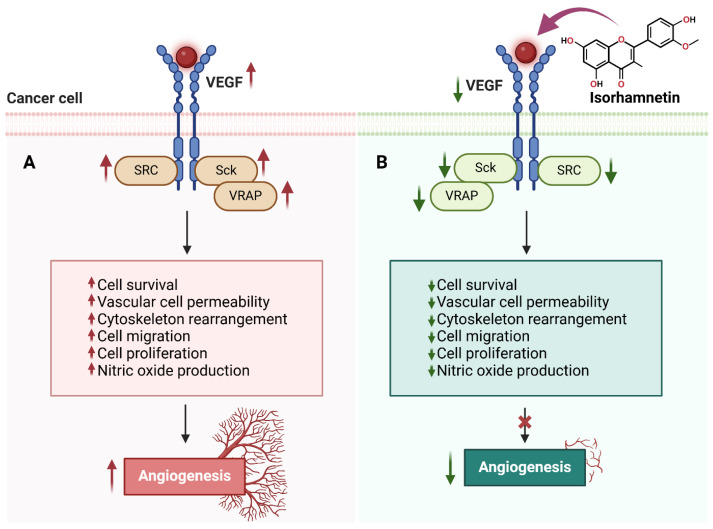
The effects of isorhamnetin on VEGF signaling and angiogenesis in cancer cells. (**A**) Upregulated VEGF signaling activates SRC, Sck, and VRAP in cancer cells, leading to increased cell survival, vascular cell permeability, cytoskeleton rearrangement, cell migration, cell proliferation, nitric oxide production, and angiogenesis. (**B**) Isorhamnetin inhibits VEGF signaling, resulting in the downregulation of SRC, Sck, and VRAP activities. This suppression reduces cell survival, vascular permeability, cytoskeletal changes, migration, proliferation, and nitric oxide production, ultimately blocking angiogenesis [95,99,100,101]. The figure was prepared using Biorender.

Angiogenesis (new blood vessel formation) and metastasis (spreading cancer cells to body organs) are crucial cancer progression processes (Figure 4) [91]. Isorhamnetin has shown its potential to disrupt both these key processes (angiogenesis and metastasis) [92], thereby avoiding tumor development and the spread of cancer to other parts of the body. VEGF is a major pro-angiogenic factor that facilitates the development of new blood vessels to increase the oxygen and nutrient supply, leading to cancer growth [93]. The inhibition of VEGF expression can restrict the formation of new blood vessels and limit the supply of oxygen and necessary nutrients for cancer cell survival, eventually leading to cell death [94]. Studies have shown that isorhamnetin has the potential to inhibit VEGF expression, suppressing angiogenesis and tumor vascularization. Isorhamnetin suppresses cancer growth and pulmonary metastases by downregulating VEGF and MMP-2 expression while upregulating endostatin, an angiogenesis inhibitor [95]. The compound also elevates immune markers IL-2 and IFN-γ, suggesting enhanced immune response. A recent study by Luo et al. reported the anti-metastatic effects of the isorhamnetin compound on the A549 cell line, which is non-small-cell lung cancer (NSCLC) [96]. Isorhamnetin treatment inhibited A549 cell proliferation in a dose and incubation-dependent manner, with substantial effects detected at concentrations of 2.5, 5, and 10 μM [96].

Isorhamnetin potentially suppressed crucial metastatic actions, including cell adhesion, invasion, and migration, mainly through downregulating matrix metalloproteinases MMP-2 and MMP-9 [57,97]. Isorhamnetin effectively influenced epithelial-to-mesenchymal transition (EMT) by enhancing the expression of epithelial marker E-cadherin and reducing mesenchymal markers such as N-cadherin, vimentin, and snail. These anti-EMT effects were facilitated by inhibition of the Akt/ERK signaling pathway, displaying the potential of isorhamnetin as a therapeutic compound to treat or restrict NSCLC progression and metastasis [96]. Furthermore, another study demonstrates the anti-invasive effects of the isorhamnetin compound on breast cancer (MDA-MB-231) cells. Isorhamnetin significantly inhibited cell adhesion, migration, and invasion by suppressing the activity and expression of MMP-2/9 [98]. Isorhamnetin also selectively blocked the phosphorylation of p38 MAPK and STAT3 without influencing ERK1/2 or JNK expressions, showing its potential in targeting metastatic processes in breast cancer through MMP regulation [98].

**Figure 5:**
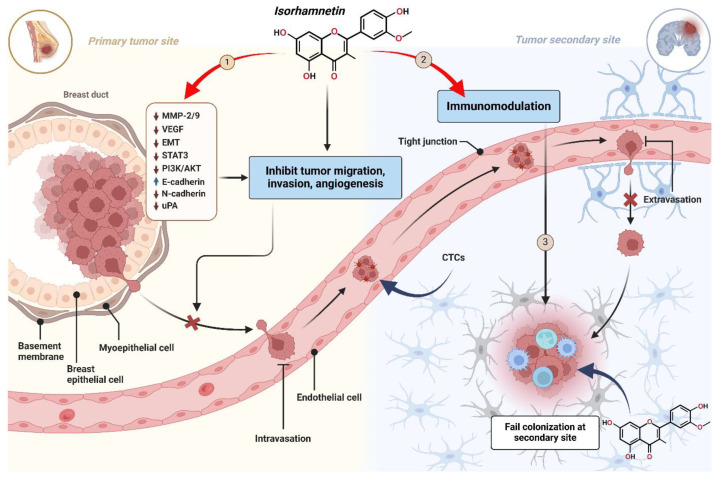
Anti-metastatic mechanism of isorhamnetin across three stages of cancer metastasis. (**1**) Primary Tumor Site: Isorhamnetin suppresses cancer cell migration, invasion, and angiogenesis by downregulating MMP-2/9, VEGF, EMT, STAT3, PI3K/AKT, and uPA. It also upregulates E-cadherin while reducing N-cadherin, preventing epithelial–mesenchymal transition (EMT) and cancer cell detachment. (**2**) Circulation Phase: Isorhamnetin enhances immunomodulation, promoting immune clearance of circulating tumor cells (CTCs). It also strengthens endothelial tight junctions, inhibiting cancer cell extravasation into distant tissues. (**3**) Secondary Tumor Site: Isorhamnetin disrupts the tumor microenvironment, inhibiting cancer cell survival, proliferation, and colonization at the secondary site [116,117,118]. The figure was prepared using Biorender.

Figure 5 describes the anti-metastatic mechanism of the isorhamnetin compound at different stages of cancer metastasis. It explains its role in preventing cancer development and colonization by taking the example of breast cancer as a primary and the brain as a secondary tumor site [102]. The three stages were considered to explain the mechanism of inhibiting metastasis using an isorhamnetin compound.

#### 3.3.1. Stage 1: Inhibition of Cancer Cell Invasion and Migration at the Primary Tumor Site

Cancer metastasis initiates with the local invasion of tumor cells into neighboring tissues [103]. Cancer cells undergo numerous alterations that increase their motility and break down the extracellular matrix (ECM) to invade. MMPs, integrins, and other proteolytic enzymes often support these changes. Isorhamnetin suppresses cancer cell migration and invasion at the primary tumor site [96]. The MMP-2 and MMP-9 are the key matrix proteins responsible for degrading the basement membrane and mainly for initiating the metastasis process [104,105,106,107,108]. It is reported that the tumor-derived proteolytically active MMP-2 is an early regulator of metastasis [109]. When isorhamnetin interacts with the cancer cell, it downregulates key molecular markers playing a key role in tumor progression, including MMP-2 and MMP-9 (matrix metalloproteinases accountable for degrading the basement membrane) [96]. The isorhamnetin compound decreased the expression levels MMP-9 without cytotoxic effects, suggesting that it can potentially be an essential natural antioxidant and MMP inhibitor related to oxidative stress [20]. Angiogenesis is a central process for tissue growth, repair, and tumor survival [110]. The VEGF promotes angiogenesis by increasing the formation of new blood vessels, which are vital in supplying oxygen and nutrients to support tumor growth and facilitate metastasis (Figure 4 and Figure 5) [110,111,112]. Isorhamnetin reduces or suppresses VEGF signaling by inhibiting the PI3K/AKT and STAT3 pathways, which are key regulators of VEGF expression (Figure 4 and Figure 5) [28,78]. By suppressing these pathways, isorhamnetin reduces VEGF production, impairing angiogenesis and eventually suppressing cancer cell metastasis [113,114]. Additionally, it stabilizes endothelial barriers, further limiting VEGF’s pro-angiogenic effect. A recent study suggests that isorhamnetin effectively decreases HSC-T6 activation, the expression of COLA1 and α-SMA, and suppresses PI3K/AKT signaling in vitro model, ultimately acting as a therapeutic compound [115].

The isorhamnetin effectively downregulated EMT (epithelial–mesenchymal transition), and STAT3 (which is a transcription factor endorsing tumor invasion) to prevent blood vessel formation. It also suppresses the PI3K/AKT signaling, eventually reducing cell proliferation/survival. A recent study explores the effect of isorhamnetin on EMT, which plays a substantial role in the pathogenesis of age-related macular degeneration [99,119]. Isorhamnetin was shown to suppress EMT in both in vivo and in vitro models [99]. EMT markers were reduced significantly, showing that isorhamnetin effectively inhibited the AKT/GSK-3β pathway, a cascade-promoting EMT. Isorhamnetin activated the Nrf2 pathway, known for its antioxidant and protective roles in cells [120,121]. Isorhamnetin suppresses EMT by activating the Nrf2 pathway and inhibiting the AKT/GSK-3β pathway, providing a potential therapeutic approach for dry age-related macular degeneration treatment [28,99]. By upregulation of E-cadherin (epithelial marker) and downregulation of N-cadherin and uPA (urokinase plasminogen activator), natural compounds (e.g., isorhamnetin) potentially prevent cancer cell detachment and invasion into neighboring body tissues [122,123,124,125]. This effectively inhibits cancer cell migration and intravasation into blood vessels, as shown in Figure 5. Rho family GTPases, including RhoA, Rac1, and Cdc42, are critical for influencing cytoskeletal dynamics. Isorhamnetin has the potential to modulate the activity of these GTPases, which leads to the reduction in cell migration and invasion by stabilizing the cytoskeleton and preventing lamellipodia and filopodia formation [86,126,127,128]. Isorhamnetin promotes glucose uptake by enhancing GLUT4 translocation by activating several signaling pathways in skeletal muscle cells and possesses advantageous roles for sustaining glucose homeostasis by inhibiting hyperglycemia at physiological concentrations [86]. Collectively, these changes decrease the capability of cancer cells to invade neighboring tissues and inhibit the migration toward secondary sites, thus halting the initial steps of metastasis (Figure 5).

#### 3.3.2. Stage 2: Enhancement of Immune-Mediated Clearance of Circulating Tumor Cells (CTCs) by Isorhamnetin

Once tumor cells manage to invade the neighboring tissue, they enter the lymphatic system or bloodstream, where they become CTCs [129,130,131]. These CTCs can evade immune surveillance and cause tumor formation at the secondary site (Figure 5) [131,132]. A crucial aspect of preventing metastasis is the immune system’s ability to recognize and eliminate these CTCs [133,134,135]. Isorhamnetin has been shown to enhance the activity of immune cells, including natural killer (NK) cells and macrophages [136,137,138]. A study reported that isorhamnetin significantly enhanced TLR2/4 expression levels and the number of NKp46+ cells (in vivo) and affected innate immune responses linked with protection, signifying that isorhamnetin enhanced innate immune potency [139]. Isorhamnetin inhibits various transcriptional factors, which play a key role in modulating differentiation, proliferation, and activation of immune cells and enhancing T-cell generation [140]. The umbilical cord blood NK cell proliferation was promoted at 41.03% ± 0.48% to 67.22% ± 0.68% when isorhamnetin was present [141]. Isorhamnetin modulates multiple immune system processes and can be used for therapeutic purposes [142].

These immune cells play essential roles in the surveillance and elimination of CTCs [138]. Isorhamnetin activates NK cells by enhancing the expression of activating receptors, such as NKG2D and NKp46, which recognize and kill CTCs [143]. A study showed that isorhamnetin regulates NK cells’ activation, maturation, and killing functions [143]. Furthermore, macrophages are also stimulated by isorhamnetin to release pro-inflammatory cytokines, such as TNF-α and IL-6, which can facilitate immune response against CTCs [18,144]. Isorhamnetin at concentrations of 20 and 40 μM significantly decreases the proliferation of BEAS-2B cells stimulated with TNF-α. It also notably reduced the expression of key pro-inflammatory cytokines (IL-1β, IL-6, IL-8, and CXCL10). Furthermore, isorhamnetin (10 μM) effectively inhibited the migration induced by TNF-α [144]. Additionally, isorhamnetin treatment suppressed the phosphorylation of key proteins in the MAPK and NF-κB signaling pathways, which were activated by TNF-α, suggesting its potential to modulate these pathways in inflammatory responses [144].

Isorhamnetin might also play a role in inducing immunogenic cell death (ICD) in cancer cells [28,145]. ICD triggers the release of damage-associated molecular patterns (DAMPs) signals to alert the immune system [146]. DAMPs (HMGB1, calreticulin, and ATP) trigger the activation of dendritic cells, leading to the appearance of tumor antigens and the initiation of adaptive immune responses, including CTCs. Isorhamnetin also regulates immune checkpoints, such as PD-L1, which are generally upregulated on cancer cells to prevent immune attacks [147]. By downregulating PD-L1 expressions, isorhamnetin improves the cytotoxic effects of T cells against CTCs [148]. Studies showed that isorhamnetin influenced the immune microenvironment by activating the PTEN/PD-L1 axis [148,149,150]. Through these processes, isorhamnetin can enable the recognition and clearance of circulating tumor cells by the immune system, thereby preventing the possibility of metastasis to distant organs.

#### 3.3.3. Stage 3: Prevention of Colonization and Survival at Secondary Tumor Sites by Isorhamnetin

If CTCs reach distant organs, they must survive the hostile microenvironment to successfully colonize and start secondary tumors (Figure 5) [151]. These steps include evading anoikis (a form of cell death caused by detachment from the extracellular matrix) and adjusting to the new tissue environment, often through angiogenesis and the ability to create a supportive position. Isorhamnetin has been found to increase the susceptibility of tumor cells to anoikis [152], a form of apoptosis that occurs when detached from the ECM [57]. By regulating integrin and downstream survival pathways (PI3K/Akt and FAK) [28,99,153], isorhamnetin decreases cancer cell survival upon detachment, making it less likely for CTCs to thrive at secondary sites [153,154]. For metastatic colonies to grow, the angiogenesis is critical. Isorhamnetin inhibits angiogenesis by suppressing the expression of VEGF and MMPs, which are essential for forming blood vessels [95,155,156]. This reduces the ability of secondary tumors to begin a vascular supply and limits their survival (Figure 5) [20,156].

### 3.4. Antioxidant and Anti-Inflammatory Effects of Isorhamnetin

The antioxidant and anti-inflammatory properties of isorhamnetin are central to its anticancer capability [20]. Oxidative stress is a key cancer driver caused by an imbalance between ROS and antioxidants.

#### 3.4.1. Reduction in Endogenous ROS and RNS Levels by Isorhamnetin

Isorhamnetin reduces ROS and reactive nitrogen species (RNS) levels through numerous mechanisms, mainly by acting as a direct antioxidant [90]. Isorhamnetin effectively scavenges detrimental radicals, such as superoxide (O_2_−), hydroxyl radicals (OH•), and hydrogen peroxide (H_2_O_2_), by donating electrons to neutralize them [157,158,159], eventually converting them from highly reactive species into less harmful and stable molecules. This process helps reduce oxidative stress that causes DNA damage and contributes to the development of cancers [160]. By reducing ROS and RNS, isorhamnetin helps protect cells from oxidative impairment. Research has proved that pretreatment with isorhamnetin inhibits the formation of ROS and mitigates GSH depletion induced by t-butyl hydroperoxide. This results in reduced ROS levels and a subsequent decrease in t-butyl hydroperoxide-induced cell death [161]. Furthermore, isorhamnetin enhances the phosphorylation of key signaling molecules such as ERK1/2, PKCδ, and AMPK, indicating its protective role against oxidative stress in hepatocytes [90]. Another investigation found that isorhamnetin-induced heme oxygenase-1 (HO-1) expression led to diminished ROS production, and its antioxidant properties may play a crucial role in suppressing COX-2 expression, thereby potentially reducing inflammation [157]. Isorhamnetin has shown promising effects on glucose uptake in skeletal muscle cells at low concentrations (1 nM). It promotes GLUT4 translocation to the plasma membrane in L6 myotubes, mainly by stimulating the JAK2/STAT signaling pathway [162]. It indicates that isorhamnetin is a therapeutic agent for improving glucose homeostasis and is involved in glucose uptake [86].

In addition to its direct scavenging ability, isorhamnetin triggers the action of several key endogenous antioxidant enzymes, such as superoxide dismutase (SOD), glutathione peroxidase (GPx), and catalase (CAT) [163]. These endogenous antioxidant enzymes play vital roles in maintaining cellular redox balance by altering highly reactive ROS into less reactive or non-toxic molecules [164,165,166]. For example, SOD catalyzes the conversion of superoxide into hydrogen peroxide. At the same time, CAT and GPx enzymes play a key role in further breaking down hydrogen peroxide into water and oxygen, eventually reducing oxidative damage [167,168]. The ability of isorhamnetin to upregulate these antioxidant defenses strengthens the ability of cells to cope with oxidative stress from any external stimuli or environmental changes. Isorhamnetin exerts a protective effect by reducing the inflammatory response, reducing oxidative stress, improving endothelial function, and inhibiting apoptosis by activating the PI3K/AKT/eNOS pathway [166].

Furthermore, isorhamnetin triggers the Nrf2 signaling pathway, an essential regulator of the cellular antioxidant response [90,99,169]. Studies have shown that isorhamnetin increased Nrf2 activity and target gene expression [78,90]. Under cellular oxidative stress, Nrf2 dissociates from its inhibitor, Keap1, and is translocated to the nucleus, upregulating the expression of antioxidant genes [170]. This leads to the upregulation of numerous antioxidant proteins, such as SOD, CAT, and GPx, increasing the overall antioxidant capacity of cells [171]. Isorhamnetin also inhibits the activity of ROS-producing enzymes, such as NADPH oxidase (NOX) and cyclooxygenase-2 (COX-2), both of which contribute to ROS production [171]. By downregulating these enzymes, isorhamnetin decreases the additional generation of ROS, further mitigating oxidative stress [172,173]. Additionally, isorhamnetin stabilizes mitochondrial function, a source of cellular stress, thereby reducing mitochondrial ROS generation [174]. In this way, isorhamnetin helps preserve mitochondrial integrity and prevents oxidative damage, eventually inhibiting cancer progression [38]. Through these combined actions, isorhamnetin maintains redox homeostasis and protects against oxidative stress [175].

Isorhamnetin plays a role in immunomodulation, enhancing the immune system’s ability to eliminate circulating tumor cells [176]. By tightening endothelial junctions in blood vessels, isorhamnetin prevents cancer cells’ intravasation, circulation, and extravasation into distant tissues [177]. This step is critical to halting the spread of tumor cells to secondary sites (Figure 5). Isorhamnetin triggers the Nrf2 signaling pathway and regulates oxidative stress and inflammatory responses [89,175]. Inflammation and immune cell infiltration play an essential role in cancer cell migration and extravasation in the tumor microenvironment [78]. A study investigates the effects of saffron petal extract (containing isorhamnetin compound) on inflammation and oxidative stress in a co-culture model of human intestinal cells and macrophages, simulating the inflammatory bowel disease environment. Results suggest that saffron petal extract (containing isorhamnetin compound) has potential as a complementary therapeutic approach due to its anti-inflammatory and antioxidant properties [178].

#### 3.4.2. Kappa-Light-Chain-Enhancer of Activated B Cells (NF-κB) and Cyclooxygenase-2 (COX-2) Inhibition by Isorhamnetin

The NF-κB pathway is a key regulator of inflammation and cell survival [179,180]. Isorhamnetin has been shown to inhibit NF-κB activation, thus decreasing the expression of pro-inflammatory cytokines and facilitating apoptosis in cancer cells [181,182]. Furthermore, isorhamnetin inhibits COX-2, an enzyme that plays a vital role in the inflammatory response and the production of prostaglandins, which are implicated in cancer progression. A recent study showed that isorhamnetin ameliorated PGE2, IL-1β, and IL-6 levels and decreased COX-2 and TNF-α expression [181]. Isorhamnetin exhibited antioxidant, hypoglycemic, and anti-inflammatory properties in in vitro and in vivo models [183]. Isorhamnetin inhibited hematobiochemical dysregulation and AChE/BChE/COX2/NOx signaling in diabetic rats. Molecular docking profiles discovered strong interaction and stability of isorhamnetin for targeting AChE/BChE/COX2/NOx [183]. A study by Alqudah et al. showed that Isorhamnetin ameliorates insulin resistance, oxidative stress, and inflammation [184]. Isorhamnetin shows significant potential as a hypoglycemic agent for managing type 2 diabetes (T2D) due to its multifaceted effects. These include reducing insulin resistance, enhancing glucose uptake in skeletal muscle, improving lipid metabolism, lowering oxidative stress and inflammation, and activating the GLUT4-AMPK pathway [184]. The mechanisms and effects of isorhamnetin closely resemble those of metformin [185]. Through these mechanisms, isorhamnetin reduces oxidative stress and inflammation, two major contributors to cancer development.

**Figure 6:**
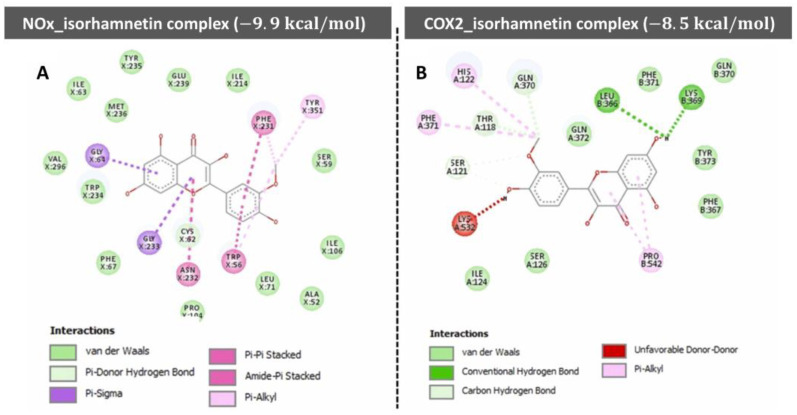
The molecular docking profile of compounds (i.e., isorhamnetin) in the docked cavity of (**A**) nitric oxide synthase and (**B**) COX2. Reprinted with permission from [183].

Figure 6 demonstrates the molecular docking interactions of isorhamnetin with two proteins, NOx and COX-2, highlighting their respective binding affinities and interaction mechanisms. The NOx-isorhamnetin complex exhibits a higher binding affinity with a docking score of −9.9 kcal/mol, suggesting a strong interaction (Figure 6A). This interaction is stabilized through van der Waals forces, hydrogen bonds, and aromatic interactions such as Pi-Pi stacking and Pi-Alkyl contacts. Key residues like TYR-235, PHE-231, and ILE-234 are crucial in anchoring isorhamnetin within the binding pocket.

In contrast, the COX-2-isorhamnetin complex shows a slightly weaker binding affinity, with a score of −8.5 kcal/mol (Figure 6B). Van der Waals interactions, hydrogen bonds, and hydrophobic contacts with residues like LEU-386, GLN-370, and TYR-371 facilitate stabilization in this complex. An unfavorable interaction with LYS-532 slightly reduces the stability of this complex. Overall, the NOx complex demonstrates stronger binding interactions than the COX-2 complex, indicating that isorhamnetin may exert more potent effects on NOx-related pathways. This insight supports isorhamnetin’s potential therapeutic role through targeted molecular interactions.

### 3.5. Activation of the p53 Pathway by Isorhamnetin

**Figure 7:**
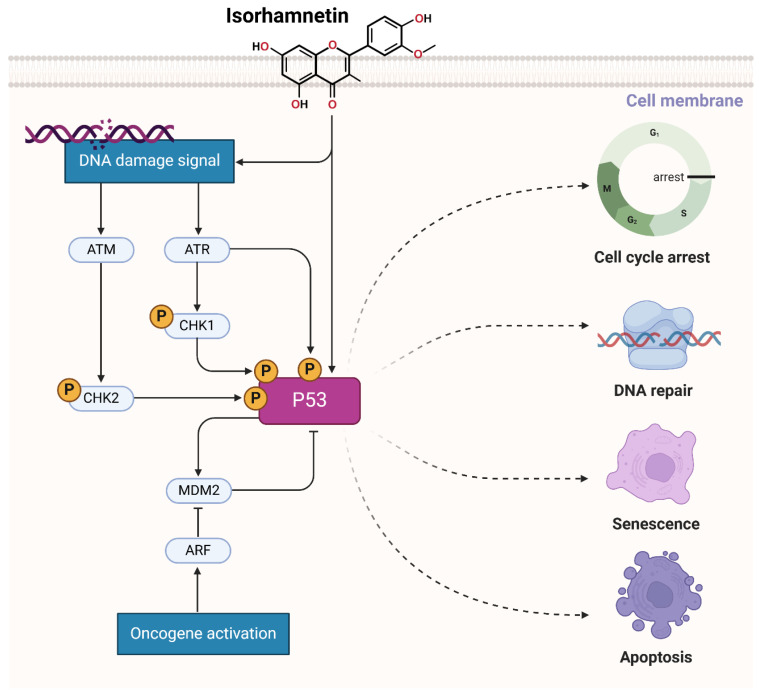
Isorhamnetin activates the ATM/ATR and p53 pathways by influencing or damaging DNA, likely through oxidative stress or cellular homeostasis disruption, generating signals for DNA damage recognition [62,193,194,195]. The figure was prepared using Biorender.

Isorhamnetin triggers the ATM/ATR pathway by inducing DNA damage, likely through oxidative stress or disruption of cellular homeostasis, which generates signals for DNA damage recognition [62,186]. This leads to the activation of ATM (Ataxia Telangiectasia Mutated) and ATR (ATM and Rad3-related) kinases, crucial regulators of the DNA damage response. These kinases phosphorylate downstream effectors, including checkpoint kinases CHK1 and CHK2, which subsequently phosphorylate the tumor suppressor protein p53. Phosphorylation of p53 stabilizes it by avoiding its degradation through MDM2 inhibition, permitting its initiation [187,188,189]. Active p53 orchestrates a multifaceted cellular response, promoting cell cycle arrest to allow DNA repair, inducing apoptosis if the damage is severe, and starting cellular senescence to prevent the proliferation of damaged cells (Figure 7) [190]. By triggering this pathway, isorhamnetin increases genomic integrity and exerts its anticancer effects, indicating its therapeutic potential as a modulator of the DNA damage response and tumor suppressor pathways [191,192]. Studies showed that isorhamnetin induces radioprotective effects. Isorhamnetin inhibited radiation-induced cell death and improved cell survival [193]. The radioprotective effect of isorhamnetin was ATM-dependent and abolished with an ATM inhibitor. In mice, isorhamnetin enhanced survival after radiation-induced gastrointestinal damage [193].

### 3.6. Activation of MAPK Pathway by Isorhamnetin

Isorhamnetin is an effective phytochemical that has gained significant attention due to its potential for biomedical applications [196,197,198]. Recent molecular studies have illuminated that isorhamnetin has the potential to modulate the mitogen-activated protein kinase (MAPK) pathway [20,99,199,200], which is a central signaling cascade for cellular responses to external stimuli, including oxidative stress, inflammation, and oncogenic signals [201,202,203]. In the MAPK signaling pathway, including the key markers (subfamilies) such as ERK (extracellular signal-regulated kinase), JNK (c-Jun N-terminal kinase), and p38 are play a central role in regulating cell functions such as proliferation [204], apoptosis [205], differentiation [206], and stress responses [207,208].

Isorhamnetin interacts with membrane receptors such as receptor tyrosine kinases (RTKs) or G-protein coupled receptors (GPCRs) upon cellular uptake [209]. This interaction stimulated a cascade of phosphorylation actions, stimulating Ras, a small GTPase protein [210,211]. Stimulated Ras later recruits and activates Raf, a serine/threonine kinase. Raf phosphorylates and activates MEK1/2, which in turn phosphorylates and activates ERK1/2. ERK1/2 translocate to the nucleus, influencing numerous transcription factors and ultimately driving gene expression in cell survival, proliferation, repair processes, and apoptosis [212,213,214]. A study showed that isorhamnetin exhibits promising therapeutic potential in gastric cancer. Network pharmacology analysis recognized MAPK14 and ERBB3 as key molecular targets, with MAPK14 showing significant upregulation and correlating with poor patient survival. Experimental data further demonstrate that isorhamnetin suppresses cell growth and migration and induces apoptosis via MAPK/mTOR pathway [215]. Studies showed that isorhamnetin can inhibit the proliferation and migration of gastric cancer cells, possibly by downregulating MAPK14 expression, which is linked to poor prognosis [216]. These results suggest that isorhamnetin’s therapeutic potential may stem from its ability to modulate the MAPK pathway, thereby reducing tumor growth and metastasis [217].

In addition to ERK activation, isorhamnetin influences the JNK and p38 MAPK branches. Under oxidative stress conditions, isorhamnetin mitigates ROS production, stabilizing the intracellular redox balance [218,219]. This antioxidant effect curtails the overactivation of JNK and p38 pathways, avoiding excessive inflammatory responses and apoptosis [220]. Interestingly, isorhamnetin selectively increases JNK and p38 activity in cancer cells, endorsing pro-apoptotic signaling [221]. Isorhamnetin accomplishes this by inducing the expression of upstream kinases, which phosphorylate JNK and p38 [222,223]. The selective cytotoxicity of isorhamnetin against malignant cells underscores its therapeutic potential as an anticancer agent. The study by Chen et al. showed that isorhamnetin activates the ERK signaling pathway and increases endogenous ROS levels, suggesting its potential as a therapeutic agent for oral squamous cell carcinoma [66]. Isorhamnetin has demonstrated protective effects against liver fibrosis by preventing the activation of hepatic stellate cells and ECM deposition, primarily mediated via downregulation of TGF-β1 and the inhibition of Smad3 and p38 MAPK signaling pathways [224]. It suggests that isorhamnetin’s action on the MAPK pathway is vital in avoiding liver fibrosis [224].

Beyond isorhamnetin’s antioxidant and apoptotic roles, it exerts potent anti-inflammatory effects by regulating MAPK-driven cytokine production [225,226]. By reducing the activation of NF-κB (a downstream effector of the MAPK pathway), isorhamnetin inhibits the transcription of pro-inflammatory cytokines such as TNF-α, IL-6, and IL-1β [227,228,229]. This dual inhibition of MAPK and NF-κB signaling by isorhamnetin alleviates inflammation and decreases the tumor-facilitating microenvironment [230,231]. The anticancer properties of isorhamnetin are further augmented by its capability to cause cell cycle arrest and apoptosis via MAPK-mediated pathways [232]. For instance, the activation of JNK by isorhamnetin improves the phosphorylation of p53, which is a tumor suppressor protein, and leads to the transcription of pro-apoptotic genes like BAX and PUMA [181,233,234,235]. Simultaneously, isorhamnetin disrupts the phosphorylation of BCL-2, an anti-apoptotic protein, tilting the cancerous cells’ balance towards programmed cell death [236].

Thus, the isorhamnetin compound is a multifaceted regulator of the MAPK pathway, showing its antioxidant, anti-inflammatory, and anticancer potential to modulate various cellular functions. By targeting specific branches of the MAPK cascade context-dependent, isorhamnetin displays notable potential as a therapeutic bioactive compound for dealing with oxidative stress, chronic inflammation, and tumor prevention. Further research might be required to unravel its precise molecular interactions and optimize its clinical applications for real-world applications.

### 3.7. Modulation of Tumor Microenvironment and Immune Response by Isorhamnetin

The cancer tumor microenvironment (TME) comprises various cell types, extracellular matrix components, and signaling molecules that affect cancer development [237]. Isorhamnetin influences the TME by targeting cancer-associated fibroblasts (CAFs) and immune cell infiltration, which play key roles in tumor growth and metastasis.

#### 3.7.1. Impact of Isorhamnetin on CAFs

CAFs are stromal cells within the TME that support tumor growth by secreting growth factors, cytokines, and extracellular matrix components [238,239,240,241,242,243,244]. Modulating CAFs presents a promising strategy to enhance the efficacy of therapies. In this review, we discussed the therapeutic potential of isorhamnetin, known for its anti-proliferative, anti-fibrotic, and anti-inflammatory properties in various cancers, by examining its effects on the tumor microenvironment, specifically CAFs. Isorhamnetin was found to suppress CAF proliferation, induce apoptosis, and cause cell cycle arrest by disrupting mitochondrial function [245]. Notably, it reduced the expression of inflammatory CAF (iCAF) markers, including IL1A, IL6, LIF, and CXCL1, while promoting a shift toward myofibroblast-like CAFs (myCAFs), as indicated by an increased presence of αSMA-positive cells [150]. Isorhamnetin showed the potential to inhibit CAF proliferation and drive a phenotypic switch from iCAFs to myCAFs, supporting its possible role in combination therapies targeting the tumor microenvironment [246,247]. Isorhamnetin inhibits the activation of CAFs, which helps reduce tumor cell proliferation, invasion, and angiogenesis [150]. Isorhamnetin effectively modulates the hypoxic tumor microenvironment in gastric cancer by targeting PI3K and inhibiting the PI3K–AKT–mTOR pathway [62,78,246]. This results in suppressed adaptive autophagy, decreased mitochondrial membrane potential, and triggers mitochondria-mediated apoptosis. Despite weaker autophagy inhibition than 3-MA, isorhamnetin validates superior efficacy in promoting apoptosis within hypoxic conditions [246], highlighting its potential as a therapeutic agent in altering the tumor microenvironment [248].

#### 3.7.2. Modulation of Immune Response by Isorhamnetin

**Figure 8:**
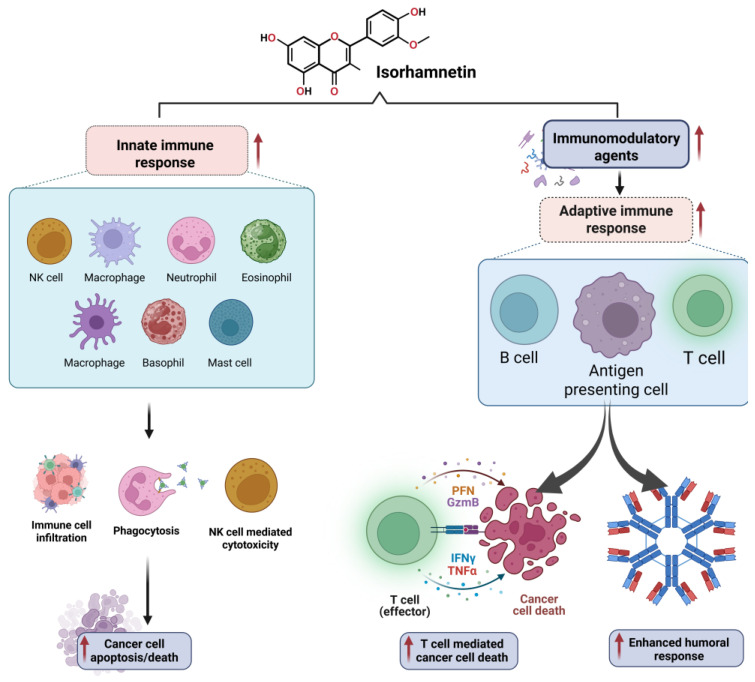
The immunomodulatory effects of isorhamnetin on the innate and adaptive immune responses. Isorhamnetin enhances the innate immune response by activating various immune cells, including NK cells, macrophages, neutrophils, eosinophils, basophils, and mast cells. This activation leads to immune cell infiltration, increased phagocytosis, and NK cell-mediated cytotoxicity, resulting in cancer cell apoptosis and death. Simultaneously, isorhamnetin stimulates adaptive immunity by modulating APCs, B cells, and T cells. Enhanced T cell activity promotes cancer cell death through effector mechanisms involving perforin (PFN), granzyme B (GzmB), interferon-gamma (IFNγ), and tumor necrosis factor-alpha (TNFα). Additionally, isorhamnetin boosts the humoral immune response, amplifying antibody production for further immune defense [19,260]. The figure was prepared using Biorender.

Isorhamnetin also regulates immune cell infiltration within the TME [249,250,251]. It has been shown to promote the recruitment of tumor-suppressive immune cells, such as natural killer (NK) cells and cytotoxic T lymphocytes, while inhibiting the infiltration of pro-tumorigenic immune cells, such as tumor-associated macrophages (TAMs) [151,252,253,254]. This immune modulation contributes to the anti-tumor effects of isorhamnetin, supporting the body’s natural defense mechanisms against cancer development. The immunomodulatory effects of isorhamnetin on the innate and adaptive immune responses are shown in Figure 8. Isorhamnetin enhances the innate immune response by activating various immune cells, including NK cells, macrophages, neutrophils, eosinophils, basophils, and mast cells. This activation leads to immune cell infiltration, increased phagocytosis, and NK cell-mediated cytotoxicity, resulting in cancer cell apoptosis and death [18,139]. Simultaneously, isorhamnetin stimulates adaptive immunity by modulating antigen-presenting cells (APCs), B cells, and T cells. Enhanced T cell activity promotes cancer cell death through effector mechanisms involving perforin (PFN), granzyme B (GzmB), interferon-gamma (IFNγ), and tumor necrosis factor-alpha (TNFα) [66,255,256,257]. Furthermore, isorhamnetin lifts the humoral immune response, increasing antibody generation for additional immune defense [258,259].

## 4. Cancer Type-Specific Effects of Isorhamnetin

**Figure 9:**
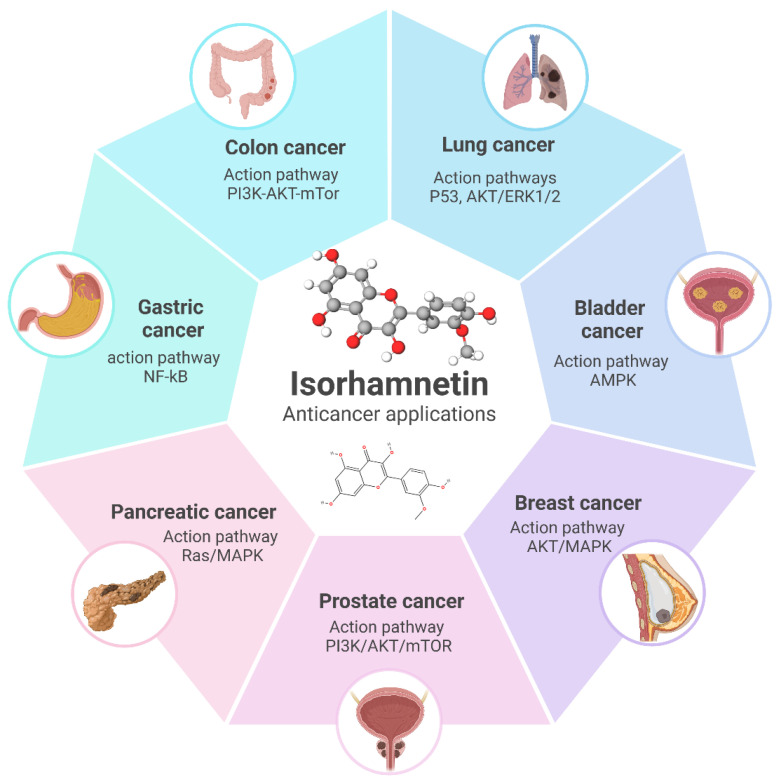
Overview of the anticancer applications of isorhamnetin and its associated molecular pathways in different cancer types. Isorhamnetin exerts its effects through key action pathways such as PI3K/AKT/mTOR in colon and prostate cancer, P53 and AKT/ERK1/2 in lung cancer, AMPK in bladder cancer, AKT/MAPK in breast cancer, NF-κB in gastric cancer, and Ras/MAPK in pancreatic cancer. These pathways highlight its potential as a versatile therapeutic agent targeting multiple mechanisms involved in cancer progression [62,96,144,166,229,269,275,276,277]. The figure was prepared using Biorender.

Isorhamnetin, a flavonoid with effective antioxidant and anti-inflammatory properties [261,262], has been shown to employ substantial anticancer effects across numerous cancer types [20,39,66,263,264,265,266], as shown in Figure 9. Its mechanisms of action, including modulation of the cell cycle (Figure 2), triggering apoptosis (Figure 3), suppression of metastasis (Figure 5), and reduction in oxidative stress, offer therapeutic opportunities in the treatment of different types of cancers [18,20,88,267]. Below, we discuss the specific effects of isorhamnetin in various cancer types.

Isorhamnetin has shown significant anticancer efficacy across various types of cancer owing to its multifaceted mechanisms of action (described in Section 3) and comprehensively summarized in Table 1. In breast and colon cancer, isorhamnetin inhibits cell proliferation by inducing cell cycle arrest (G1/S phase in breast cancer and G_0_/G_1_ phase in colon cancer) and promotes apoptosis through the activation of the mitochondrial apoptotic pathway [62,255,268,269,270]. This involves the upregulation of pro-apoptotic proteins like Bax and caspases and the downregulation of anti-apoptotic proteins such as Bcl-2 [18,271,272,273]. An in vitro and in vivo model showed that isorhamnetin plays a crucial role in Tsoong by downregulating Hsp70 gene expression and promoting apoptosis in colon cancer cells, primarily through its ability to inhibit Hsp70 [267]. Isorhamnetin treatment induces cell cycle arrest in the G2/M phase. Isorhamnetin induced cell death in vitro, as evidenced by increased phosphatidylserine exposure (48%), membrane permeabilization (30%), and nuclear condensation (54%) compared to control cells. Additionally, the Bax/Bcl-2 ratio was elevated, and a 63% loss of mitochondrial membrane potential was observed in colon cancer after isorhamnetin treatment [274]. It also suppresses EMT by targeting factors like Snail, Twist, and Vimentin, thereby reducing the metastatic potential in both breast and colon cancer types. Additionally, isorhamnetin suppresses NF-κB activity in colon cancer, leading to decreased inflammation and tumor progression, and limits the secretion of MMPs and angiogenesis, further inhibiting invasion and metastasis (Figure 4 and Figure 5).

In liver and lung cancers, isorhamnetin employs its anticancer effects by cell cycle arrest (G1/S in liver and G1 in lung cancer) and inducing apoptosis through the mitochondrial pathway, accompanied by caspase activation [231,278]. Both cancers show suppression of the PI3K/Akt/mTOR pathway, a critical pathway for cell survival and proliferation, by isorhamnetin, leading to tumor growth inhibition and enhanced cancer cell death [78,279]. Isorhamnetin also inhibits angiogenesis and metastasis by downregulating VEGF and MMP-2/9 expression in breast, liver, and lung cancers [95,280]. Furthermore, isorhamnetin suppresses the NF-κB pathway activation, which decreases pro-inflammatory cytokines such as IL-6 and TNF-α and decreases EMT, avoiding cancer cell migration and metastasis [156,251].

In gastric and prostate cancers, isorhamnetin similarly targets key pathways [281]. In both cancer types, isorhamnetin induces G0/G1 cell cycle arrest through the downregulation of cyclins and CDKs and triggers apoptosis by the mitochondrial pathway, with increased Bax and caspase expression and reduced Bcl-2 levels. A study showed that isorhamnetin induces antitumor effects in gastric cancer through modulation of the PPAR-γ activation pathway [281]. In prostate cancer, isorhamnetin influences androgen receptor signaling, inhibiting cancer cell proliferation and suppressing metastasis by inhibiting EMT and MMP activity [282,283,284]. It inhibits NF-κB activity in gastric cancer, reducing inflammation, cancer cell survival, and metastasis while showing anti-angiogenic properties [40].

In pancreatic and bladder cancers, isorhamnetin reveals its potential by inducing G0/G1 cell cycle arrest, activating the mitochondrial apoptotic pathway (intrinsic and extrinsic pathways), and inhibiting angiogenesis via VEGF suppression [155,285,286]. It also decreases metastasis in both cancers by suppressing EMT and inhibiting MMP activity. In pancreatic cancer, it inhibits the PI3K/Akt/mTOR pathway, a central driver of drug resistance and tumor growth, and enhances sensitivity to chemotherapy (Table 1). Similarly, isorhamnetin diminishes tumor development and metastasis in bladder cancer by inducing apoptosis and targeting EMT-related factors like Snail and Twist [121,177,287]. The comprehensive anticancer effects of isorhamnetin, as detailed in Table 1, highlight its ability to modulate multiple pathways that are mainly involved in cancer progression, including cell cycle regulation, apoptosis induction, inflammation suppression, and inhibition of angiogenesis and metastasis. These versatile mechanisms make isorhamnetin a capable therapeutic agent for various biomedical applications, especially against cancers, as a standalone treatment or combined with existing therapies.

**Table 1:** Overview of anticancer effects of isorhamnetin. (arrows “↑” indicates increase or activation, “↓” indicates decrease or inhibition, and “↔” indicates no significant change).

Type	Targeted Pathway	Isorhamnetin Concentration	Main Findings	Ref.
Breast cancer (in vitro)	Akt/mTOR and MEK/ERK signaling pathways	IC50: ~10 µM	↑ Apoptosis, ↑ Bax, ↑ cleaved caspase-3 ↓ Proliferation, ↓ Bcl-2, ↓ Akt, ↓, mTOR, ↓ MEK1/2, and ↓ ERK1/2 signaling	[288]
Canine mammary tumors (in vitro and in vivo)	EGFR-STAT3-PD-L1 signaling pathway	10 µM, 20 µM, and 40 µM	↑ Caspase-3, ↑ Apoptosis ↓ EGFR, ↓ STAT3, ↓ PD-L1, ↓ Migration, ↓ Invasion, ↓ Ki-67	[118]
Gastric cancer (in vitro and in vivo)	PI3K/Akt signaling pathway	IC50: ~50 µM	↑ Mitochondrial apoptosis, ↑ Caspase-3, ↑ Apoptosis. ↓ Proliferation, ↓ Invasion, ↓ Metastasis, ↓ SRC, ↓ AKT1, ↓ EGFR, ↓ PI3K/Akt,	[289]
Lung cancer (in vitro)	PI3K-Akt signaling pathway	-	↑ Apoptosis, ↑ G1 Arrest ↓ Migration, ↓ Invasion, ↓ p-PI3K, ↓ p-AKT, ↓ PI3K/Akt pathway	[290]
Prostate Cancer (in vitro)	PI3K/Akt/mTOR signaling pathway	5 µM, 10 µM, and 20 µM	↑ Apoptosis (intrinsic) ↓ Proliferation, ↓ Migration, ↓ Invasion, ↓ PI3K/Akt/mTOR, ↑ E-cadherin, ↓ Vimentin, ↓ N-cadherin, ↓ MMP-2/9	[85]
Hepatocellular carcinoma (in vivo)	Akt, MAPKs, and Nrf2 signaling pathways; PPAR-γ activation	100 mg/kg body weight (in vivo dose)	↓ Pro-inflammatory cytokines, ↓ Nrf2, ↓ Akt, ↓ MAPK, ↑ PPAR-γ, ↑ Autophagy, ↑ Apoptosis, ↑ G1 Arrest	[291]
Melanoma (in vitro)	-	IC50: 8.26 μg/ml	↑ Apoptosis, ↑ Sub-G0/G1 Arrest, ↓ S Phase, ↓ G2/M Phase, ↓ BCL-2, ↑ Bax, ↑ Caspase-3/9, ↑ DNA Fragmentation	[292]
Lung cancer (in vitro)	NF-κB signaling pathway and IL-13-mediated apoptotic mechanisms	20 µM	↑ Radiosensitivity, ↓ NF-κB, ↑ Apoptosis, ↑ Mitochondrial dysfunction, ↑ IL-13	[293]
Melanoma (in vitro and in vivo)	PI3K/Akt and NF-κB pathways, with involvement of PFKFB4	10–100 μmol/L	↓ Proliferation, ↓ Migration, ↓ Colony formation, ↑ Bax, ↑ Caspase-3, ↓ BCL-2, ↓ PI3K/Akt, ↓ NF-κB, ↓ PFKFB4, ↑ Apoptosis	[294]
Oral cancer (in vitro and in vivo)	Glycolysis signaling pathway, explicitly targeting HK2	0.1–30 μM	↓ Proliferation, ↓ Glycolysis, ↓ HK2, ↓ Ki-67, ↓ Tumor growth, ↔ PFK, ↔ PKM2	[246]
Stomach adenocarcinoma (in vitro)	MAPK/mTOR signaling pathway	20 μM, 30 μM, 40 μM, and 60 μM	↓ Proliferation, ↓ Migration, ↓ Colony formation, ↑ Apoptosis, ↑ G2/M Arrest, ↓ MAPK14, ↓ MAPK/mTOR, EMT modulation	[215]
Colorectal adenocarcinoma (in vitro and in vivo)	Apoptosis (Caspase-9 and Bcl-2)	-	↑ Apoptosis, ↑ ROS, ↑ G0/G1 Arrest, ↓ Tumor growth, ↑ Caspase-9, ↑ Hdac11, ↑ Bai1, ↓ Bcl-2	[295]
Ovarian cancer (in vitro and in vivo)	ESR1-mediated signaling pathways.	5 μM, 10 μM, 15 μM, and 20 μM	↓ Proliferation, ↓ Migration, ↓ Invasion, ↓ Ki-67, ↓ MMP-2, ↓ MMP-9, ↓ Tumor volume/weight, Targeting ESR1	[296]
Gastric cancer (in vitro and in vivo)	Mitochondria-dependent apoptosis pathway	20 µM	↑ Caspase-3, ↑ Cytochrome c, ↓ Mitochondrial membrane potential, ↑ ROS, ↑ Mitochondrial dysfunction, ↓ Migration, ↓ Proliferation, ↓ Tumor size (time & dose dependent)	[297]
Bladder cancer (in vitro and in vivo)	PPARγ/PTEN/AKT signaling pathway	10 μM, 50 μM, and 100 μM (in vitro); 5 mg/kg (in vivo)	↓ Proliferation, ↓ Tumorigenicity, ↓ G0/G1 → S transition, ↑ PPARγ/PTEN, ↓ AKT, ↓ CA9, ↑ Apoptosis, ↓ Tumor growth, ↓ Ki67	[155]
Colorectal cancer (in vitro)	ROS-mediated apoptosis and anti-inflammatory pathways	5–150 μM	↓ Mitochondrial, ↓ Metabolic, ↓ Lysosomal activity, ↑ ROS, ↓ IL-8, ↓ Proliferation, ↑ Apoptosis, ↑ Cell cycle disruption (≥100 μM)	[298]
Lung cancer (in vitro)	Akt/ERK-mediated epithelial-to-mesenchymal transition (EMT)	2.5, 5, and 10 μM	↓ Proliferation, ↓ Adhesion, ↓ Invasion, ↓ Migration, ↓ MMP-2/9, ↑ E-cadherin, ↓ N-cadherin, ↓ Vimentin, ↓ Snail, ↓ Akt/ERK, EMT reversal, ↓ Metastasis	[96]
Breast cancer (in vitro)	p38 MAPK and STAT3 signaling pathway	-	↓ Adhesion, ↓ Migration, ↓ Invasion, ↓ MMP-2/9, ↓ p38 MAPK, ↓ STAT3, ↔ ERK1/2, ↔ JNK, ↔ uPA	[98]
Breast cancer (in vitro and in vivo)	AMPK/mTOR/p70S6K signaling, ROS generation, G2/M cell cycle arrest, apoptosis pathway	10, 20, 30, 50 μM	↑ Apoptosis, ↑ G2/M Arrest, ↓ CDK1/Cyclin B1, ↑ ROS (×6.78 times), ↑ DNA damage, ↑ AMPK, ↓ mTOR/p70S6K, ↓ Proliferation	[62]
Endometrial cancer (in vitro and in vivo)	Mitochondrial dysfunction, cell death receptor pathway, endoplasmic reticulum (ER) stress pathway, UPR response, MMP2/9 expression	0 μM, 20 μM, 40 μM, and 60 μM	↑ Apoptosis (mitochondrial & death receptor), ↑ ER stress pathway, ↓ MMP-2/9, ↓ Metastasis, ↓ Tumor growth	[117]
Breast cancer (in vitro)	Akt/mTOR and MEK/ERK signaling pathways and cell cycle inhibition	100, 33.3, 11.1, 3.7, 1.2, 0.4 and 0 µM	↓ Proliferation, ↑ Apoptosis, ↓ Akt/mTOR, ↓ MEK/ERK, ↑ Akt & MEK activation (EGF reversal)	[288]
Colorectal cancer (in vitro)	HIF-1α, ROS, Nrf2, glucose transporter 1, lactate dehydrogenase A, pyruvate dehydrogenase kinase 1, heme oxygenase-1, COX-2	3, 10, 30, 69 µM	↓ HIF-1α (CoCl_2_, hypoxia, H_2_O_2_-induced), ↓ Hypoxia genes, ↓ ROS, ↓ Migration, ↓ Invasion, ↑ Nrf2, ↑ Antioxidant proteins	[299]
Gastric cancer (in vitro)	PI3K–AKT–mTOR signaling pathway	20, 40, 80, 160, and 320 µM/L	↓ Autophagy (under hypoxia), ↓ Proliferation, ↓ Mitochondrial membrane potential, ↑ Mitochondrial apoptosis, ↓ PI3K/Akt/mTOR, ↑ Apoptosis (vs. 3-MA)	[246]
Colon cancer (in vitro)	Apoptosis, cell cycle regulation, mitochondrial	50 µg/mL and 100 µg/mL	↑ G2/M Arrest, ↑ Bax/Bcl-2 ratio, ↑ Apoptosis (mitochondrial), ↑ ROS, ↑ Caspase-dependent cell death	[274]
Colon cancer (In vitro and in vivo)	Apoptosis, Hsp70 inhibition	-	↑ Apoptosis, ↓ Hsp70, ↑ Apaf1, ↑ Caspase-3/9, ↓ Tumor growth (colon cancer model)	[267]
Gastric cancer (In vitro and in silico)	MAPK/mTOR signaling pathway	20 µM and 30 µM	↓ Proliferation, ↓ Migration, ↑ Apoptosis, ↑ MAPK/mTOR activation (apoptosis induction)	[215]

## 5. Synergistic and Adjuvant Roles of Isorhamnetin for Biomedical Applications

Given the limited research on this topic, we have explored both cancer and non-cancer studies to highlight the potential of isorhamnetin for use in combination therapies, emphasizing it as a promising area for future research (Table 2). In recent investigations, isorhamnetin has shown significant potential as an adjuvant therapy due to its capability to enhance the efficacy of conventional cancer treatments (e.g., commercial drugs, radiations, nanoparticles (NPs), other natural compounds, etc.), and act synergistically to achieve improved outcomes in biomedical applications [300]. Isorhamnetin sensitizes tumor cells to therapeutic interventions through a combination of biochemical and molecular mechanisms, increasing the effectiveness of treatments while diminishing harmful or undesired effects.

**Figure 10:**
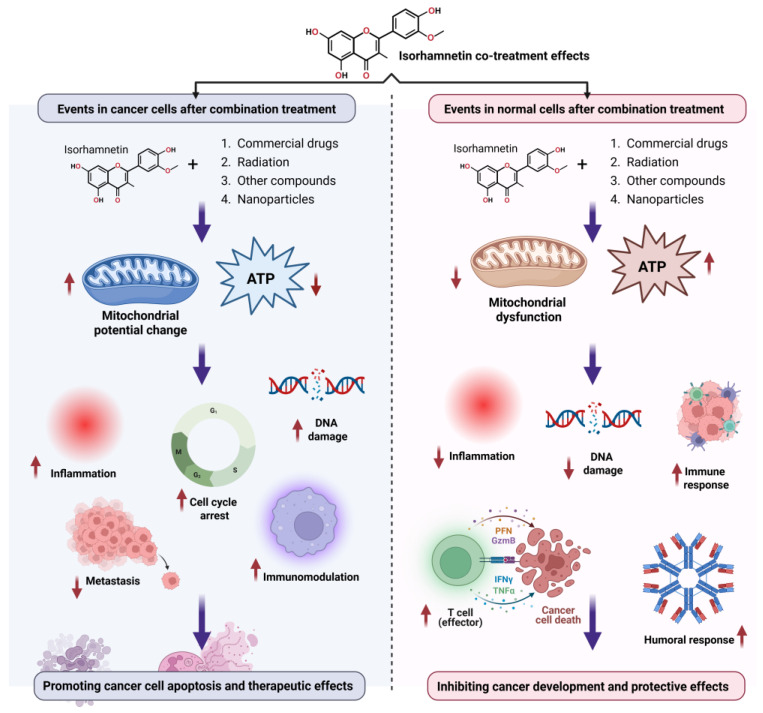
The overview of the major effects of isorhamnetin in combination with commercial drugs, radiation, and other natural compounds shows its probable role in tumor suppression and its protective effects in healthy tissues.

Chemotherapy and radiotherapy remain the cornerstone treatments for many types of cancer, but their effectiveness is often limited by undesired toxicity, cancer cell resistance, and damage to healthy tissues [301,302,303,304]. Isorhamnetin bioactive compound sensitizes tumor cells to therapeutic interventions, modulation of oxidative stress, eventually leading to improved cell death and selectivity in combination treatments [38,62,255,305]. In chemotherapy, isorhamnetin enhances the cytotoxic effects of cisplatin, doxorubicin, and paclitaxel by influencing key pathways that regulate cell survival and apoptosis. For example, it inhibits the PI3K/Akt/mTOR pathway, often upregulated in chemoresistant tumor cells, thereby enhancing the apoptotic response. Additionally, isorhamnetin targets numerous signaling pathways vital for tumor progression and resistance. By influencing endogenous ROS levels, modulating key survival and apoptotic pathways, altering the tumor microenvironment, and improving drug retention, isorhamnetin is an effective sensitizer to cancer therapies. When combined with commercial drugs, isorhamnetin enhances therapeutic efficacy by targeting multiple pathways (described in Section 3). The combination induces mitochondrial dysfunction in cancerous cells, disrupting mitochondrial membrane potential, cytochrome c release, and ensuing apoptosis (Figure 10). ATP production in cancer cells is meaningfully reduced, impairing their energy-dependent processes. Additionally, isorhamnetin and commercial anticancer drug combinations cause DNA damage, overwhelming the cancer cell repair mechanisms and leading to cell cycle arrest at critical checkpoints, such as G1 or G2/M phases. These effects are coupled with immunomodulatory actions that activate immune cells to eliminate cancer cells effectively. In healthy cells, the combination with drugs is protective by enhancing mitochondrial function and modulating inflammation, reducing the risk of off-target toxicities. The study showed that combining isorhamnetin with cisplatin and carboplatin improves anticancer effects. The synergistic effects were observed, and reduced tumor cell survival was noted compared to the cisplatin and carboplatin drugs alone [306]. Furthermore, the combination of isorhamnetin, cisplatin, and carboplatin drugs exhibits more potent inhibition of cancer cell migration, cell cycle arrest, and mitochondrial dysfunction, contributing to the increased efficacy of the combination therapy [306]. Isorhamnetin combined with doxorubicin significantly increases doxorubicin-induced apoptosis in breast cancer, boosting the apoptosis rate from 5.83% (with doxorubicin alone) to 35.38% (isorhamnetin + doxorubicin) [268]. It induces G2/M cell cycle arrest by influencing the CDK1/Cyclin B1 complex and promotes a 6.78-fold increase in endogenous ROS production, leading to DNA double-strand breaks. When combined with doxorubicin, isorhamnetin significantly inhibits tumorigenesis and is considered a favorable candidate for combination treatment in breast cancer [268].

Simultaneously, isorhamnetin protects healthy cells or tissues from oxidative stress by enhancing their antioxidant capacity. These dual effects make isorhamnetin a promising adjuvant to conventional therapies. Isorhamnetin has been shown to improve the antidepressant effects of escitalopram, demonstrating a reduction in floating time in the forced swim test [54]. This bioactive compound also restores key neuroprotective markers (Nrf2, BDNF, and HO-1 levels), enhancing behavioral outcomes. Moreover, isorhamnetin induces synergistic effects, thereby boosting the efficacy of conventional antidepressant therapies [54]. A recent study showed that isorhamnetin significantly suppresses the cytotoxic effects of cisplatin, inhibiting both cisplatin-induced apoptosis and inflammatory responses [38].

In combination with radiation, isorhamnetin sensitizes cancer cells to oxidative stress. The interaction amplifies ROS generation, particularly in cancers, causing DNA strand breaks and impairing replication. Tumor cells exhibit a reduced ability to repair radiation-induced DNA damage, leading to apoptosis or mitotic catastrophe. Moreover, mitochondrial dysfunction further compromises cancer cell survival during radiation therapy. In normal cells, isorhamnetin reduces radiation-induced oxidative stress and inflammation. Modulating the expression of inflammatory cytokines such as TNF-α and IL-6 prevents radiation damage to healthy tissues. Enhanced immune responses also support the repair of normal tissues, ensuring better tolerance to radiation therapy. Isorhamnetin pretreatment enhances the radiosensitivity of cells, increasing apoptosis in lung cancer cells when combined with radiation exposure [293]. Furthermore, isorhamnetin pretreatment enhances the expression of apoptosis-related proteins [293].

Combining isorhamnetin with several NPs offers an engaging platform for targeted drug delivery and improved therapeutic outcomes [307,308,309,310]. In tumor cells, NPs coupled with isorhamnetin can carry the compound directly to the tumor microenvironment site, increasing local drug concentration and minimizing systemic toxicity [311,312,313,314]. These combinations (isorhamnetin + NPs) can induce mitochondrial dysfunction and oxidative stress in cancer cells, leading to higher apoptosis rates [315,316]. The nanoparticle-based treatment improves the cellular uptake of the isorhamnetin compound, intensifying its biomedical effects [317,318]. In healthy cells, nanoparticle-based delivery reduces off-target exposure and preserves tissue health [319]. The enhanced bioavailability of isorhamnetin also increases its anti-inflammatory and immunomodulatory characteristics in healthy tissues [313,320,321,322]. Combining the isorhamnetin compound with several NPs could be an interesting route for future exploration.

Combining isorhamnetin with other flavonoids or natural compounds has established improved effects, increasing overall biomedical events beyond what either compound attains alone [275,323,324,325,326]. For example, combining isorhamnetin with quercetin enhances apoptosis and prevents cancer cell survival and proliferation [327]. It is reported that the quercetin and isorhamnetin compounds enhance heme oxygenase 1 (HO-1) levels and contribute to the down-regulation of miR-155 [157]. Quercetin and isorhamnetin suppressed the mRNA and protein expression levels of TNF-α and inflammatory cytokines [328]. Isorhamnetin establishes synergistic effects when combined with other natural compounds. These combinations improve overall anti-inflammatory and antioxidant activities, preventing tumor-promoting effects of chronic inflammation [327]. Additionally, co-administration of isorhamnetin with other natural compounds might strengthen the immune responses and ability to identify and attack cancer cells by enhancing the activity of effector T-cells and natural killer cells (Figure 10). In healthy cells or tissues, isorhamnetin, in combination with other natural compounds, helps maintain homeostasis by endorsing tissue repair, modifying immune responses, and decreasing severe inflammation [181,248,328,329,330,331,332,333,334]. Additionally, isorhamnetin has synergized with curcumin, a polyphenol with potent anti-inflammatory and anticancer effects. Together, these compounds efficiently suppress cancer cell survival and proliferation, angiogenesis, and metastasis by targeting multiple signaling pathways [26,335,336].

Isorhamnetin combinations with chemotherapeutic drugs, radiation, NPs, or other natural compounds might deliver a multifaceted cancer treatment method. These combinations exploit vulnerabilities in tumor cells, increasing selectivity and immune activation and evasion. This dual action makes isorhamnetin-based combination treatments a promising path for targeted cancer treatments while reducing the risk of harmful or undesired effects on healthy cells.

**Table 2:** Combination treatment using isorhamnetin (in both cancer/noncancer studies).

Cancer	Combination	Main Findings in the Combination Treatment	Study Target	Ref.
No	Isorhamnetin + escitalopram	✓ Isorhamnetin enhances the antidepressant effects of escitalopram. ✓ Reduced floating time in the forced swim test. ✓ Restored Nrf2, BDNF, and HO-1 levels ✓ Improved behavioral outcomes ✓ Isorhamnetin induces synergistic effects and improves the efficacy of conventional antidepressant therapy.	Antidepression (in vivo)	[54]
Yes	Isorhamnetin + carboplatin + cisplatin	✓ Isorhamnetin enhances the anticancer effects of cisplatin and carboplatin ✓ Combination treatment significantly decreases cell viability compared to individual-drug treatments. ✓ Induced apoptosis and loss of mitochondrial membrane potential. ✓ Inhibition of cancer cell migration is higher in combination treatment. ✓ Cell cycle arrest occurred at the G2/M phase, and microtubule depolymerization was activated in the combination treatments.	Lung cancer (in vitro)	[306]
No	Isorhamnetin + cisplatin	✓ Isorhamnetin notably reduced the cytotoxic effects of cisplatin. ✓ Isorhamnetin inhibited cisplatin-induced apoptosis and inflammatory responses. ✓ Oral administration of isorhamnetin before and after cisplatin injection improved renal function and reduced kidney tubule damage.	Kidney protection (in vitro, in vivo)	[38]
Yes	Isorhamnetin + radiotherapy	✓ Isorhamnetin pretreatment enhanced radiosensitivity ✓ Increased apoptosis and collapse of mitochondrial membrane potential by isorhamnetin treatment and radiation exposure. ✓ Isorhamnetin pretreatment suppressed radiation-induced upregulation of NF-κBp65 ✓ Isorhamnetin enhanced the expression of proteins related to apoptosis. ✓ IL-13 expression was positively correlated with isorhamnetin-mediated radiosensitization	Lung cancer radiosensitization (in vitro)	[293]
No	Quercetin + Isorhamnetin + Quercetin-3-glucuronide	✓ Quercetin and isorhamnetin downregulated mRNA and protein levels of TNF-α and inflammatory cytokines (IL-1β, IL-6, MIP-1α, iNOS). ✓ Quercetin and isorhamnetin improved heme oxygenase 1 (HO-1) levels. ✓ Down-regulation of miR-155 by quercetin and isorhamnetin	anti-inflammatory effects (in vitro)	[327]
Yes	Isorhamnetin + Isorhamnetin-3-glucuronide + Quercetin	✓ The combination treatment inhibited MCF-7 cell growth ✓ Induced S-phase arrest and early-phase apoptosis. ✓ Activated ROS-dependent apoptosis pathway.	Breast cancer cytotoxic effects (in vitro)	[268]
Yes	Isorhamnetin + Doxorubicin	✓ Isorhamnetin (50 µM) enhanced DOX-induced apoptosis from 5.83% (DOX) to 35.38%. ✓ Isorhamnetin induced G2/M cell cycle arrest via modulation of the CDK1/Cyclin B1 complex. ✓ Increased ROS generation (6.78-fold), contributing to DNA damage.5 ✓ Inhibited mTOR/p70S6K signaling by AMPK activation to inhibit proliferation. ✓ Isorhamnetin combined with Doxorubicin significantly inhibited tumorigenesis	Breast cancer (in vitro, in vivo)	[62]
No	Combination + sildenafil (in vivo)	✓ Isorhamnetin improved hemodynamic parameters (mPAP, RVSP) and alleviated right ventricular hypertrophy (RVHI, CSA). ✓ Inhibited TNF-α-induced HPASMC proliferation and inflammation. ✓ Upregulated BMP signaling.	Pulmonary arterial hypertension (PAH)	[247]

## 6. Advances in Delivery Systems for Isorhamnetin for Anticancer Applications

Despite the promising anticancer properties of isorhamnetin, its clinical applications are limited by challenges related to its bioavailability, stability, and precise delivery to tumor sites [337,338,339]. Unlike earlier reviews that discussed early-stage nanocarriers [20], our review emphasizes advanced platforms, including PLGA, solid lipid nanoparticles, and biomimetic systems developed, with an emphasis on overcoming pharmacokinetic and tumor targeting challenges.

Recent advancements in drug delivery methods have been expected to overcome these limitations, increasing the therapeutic efficiency of isorhamnetin while minimizing toxicity to healthy tissues [340]. Several novel approaches, including nanoparticle-based delivery [341,342,343], liposomal formulations [344,345,346], pro-drugs [347,348,349], and targeted delivery systems [350], have shown potential in enhancing the pharmacokinetic profile of isorhamnetin.

### 6.1. Delivery Methods

**Figure 11:**
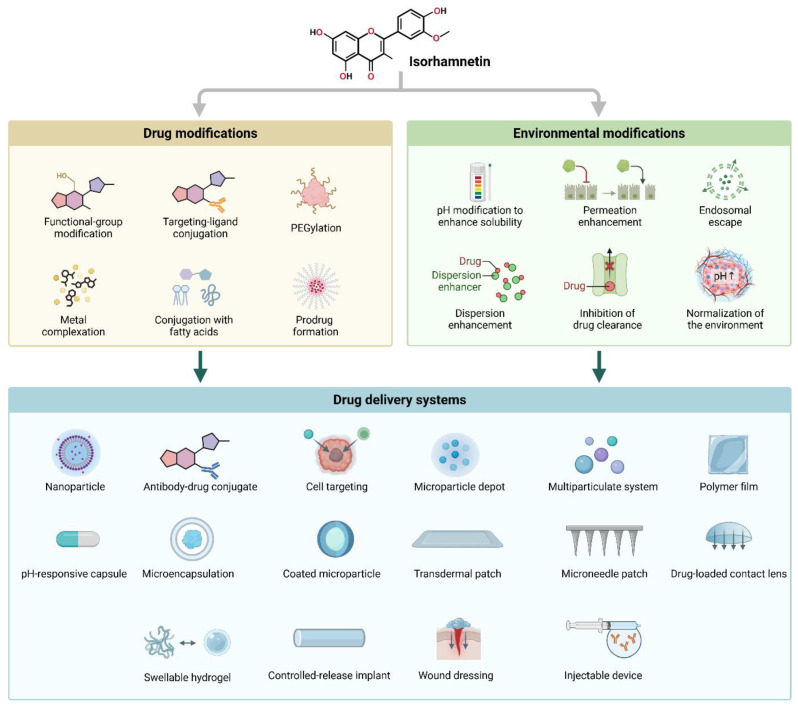
Strategies for optimizing isorhamnetin delivery. This graphical representation highlights innovative approaches to enhance the therapeutic efficacy of isorhamnetin through drug and environmental modifications. Drug modifications, such as functional-group adjustments, PEGylation, ligand targeting, and metal complexation, aim to improve solubility, permeability, and specificity while reducing off-target toxicity. Environmental modifications focus on pH adjustments, endosomal escape, permeation enhancement, and the normalization of cellular environments to optimize drug bioavailability. Together, these advancements integrate into advanced drug delivery systems, including NPs, antibody-drug conjugates, microparticles, transdermal patches, microneedles, and controlled-release implants, providing tailored and efficient therapeutic applications for isorhamnetin.

In the search for drug delivery procedures for isorhamnetin compounds, several advanced methods, as shown in Figure 11, can be employed to improve their bioavailability, stability, and targeted action [351,352,353,354]. NPs offer significant promise owing to their aptitude to encapsulate isorhamnetin, enhancing its solubility, protecting it from degradation, and allowing targeted delivery to specific targets (cells or tissues) [355,356,357]. By leveraging the exceptional characteristics of NPs, such as surface functionalization with various ligands, the isorhamnetin compound can be subjected or delivered to specific areas (Figure 11), thus increasing its therapeutic effectiveness [358,359,360].

Correspondingly, cell-targeting methods and antibody-drug conjugates permit the precise delivery of isorhamnetin to targeted diseased cells, mainly in cancer or inflammation therapy [361,362,363]. Both approaches concentrate on employing specific receptors or biomarkers on the target diseased cells (Figure 11), which could enhance the isorhamnetin efficacy while diminishing off-target or undesired effects [364,365,366].

Additional methods, such as microparticle depots and microparticle systems (Figure 11), also signify capable strategies for sustained and controlled release of the isorhamnetin compound [367,368,369]. Microparticle-based systems can carry the drug, protecting it from premature degradation and offering a slow, extended-release over time [370,371,372], mainly advantageous for chronic conditions requiring long-term treatment [373]. Polymer films and pH-responsive capsules are similar in their ability to provide controlled release [374,375,376], presenting the further advantage of protecting isorhamnetin compound release from severe body conditions [377,378], such as acidic environments in the stomach, eventually ensuring drug delivery to desired targets (Figure 11). These films and capsules dissolve in programmed conditions, warranting that isorhamnetin is carried effectively to the desired site [379].

Additionally, microencapsulation, which involves encasing isorhamnetin in micro-sized particles, shares similarities with these methods and could offer enhanced stability and release control for oral and topical applications [380]. A study reported the successful application of an innovative microfluidic device to encapsulate the isorhamnetin compound [380]. This method yielded an encapsulation efficiency of 17.92% and a loading capacity of 1.63% [380]. The encapsulation procedure depends on intramolecular interactions enabled by the hydroxyl groups on the surfaces. Additionally, the drug (isorhamnetin) exhibited a burst release of approximately 48% within the first hour, attaining a whole release within 3 h [380]. This report indicates the potential of microfluidic technology and biogenic silica as an efficient way for the precise delivery of hydrophobic compounds like isorhamnetin, opening new paths for advanced drug delivery systems and exploring new therapeutic methods. The encapsulation of isorhamnetin, a hydrophobic flavonoid, was successfully achieved and aimed to enhance its drug delivery potential. The co-administration and microencapsulation with inulin hold substantial potential for increasing the stability of isorhamnetin for its anti-inflammatory and immunomodulatory effects in the gastrointestinal environment [381], thus increasing its therapeutic effects [382].

Microneedle patches, transdermal patches, and drug-loaded contact lenses can also provide prospects for isorhamnetin delivery [383]. These approaches permit non-invasive administration, either by the skin or eye [384]. Transdermal and microneedle patches might offer a stable release of isorhamnetin for chronic conditions. In contrast, drug-loaded contact lenses can be predominantly useful in eye care, where isorhamnetin’s anti-inflammatory and antioxidant characteristics could treat ocular diseases efficiently [99,330,385,386,387]. Additionally, controlled-release implants provide a more direct approach to drug delivery, offering extended release at a constant rate, applicable for sustained therapeutic outcomes [388]. Furthermore, swellable hydrogels, which can absorb biological fluids and release drugs (isorhamnetin) in response to specific external stimuli, are also a workable option for localized and precise delivery, particularly for wound healing or topical treatments [389]. Wound dressings, including isorhamnetin, could be helpful for tissue regeneration and inflammation control owing to their antioxidant activities [390]. These dressings can deliver a direct and continuous supply of isorhamnetin to the wound site, boosting the healing process [391]. Lastly, injectable devices also offer the benefit of rapid, controlled release and targeted drug (isorhamnetin) delivery [72]. These methods, when optimized, could meaningfully increase the therapeutic outcomes of isorhamnetin in various biomedical applications. By using these targeting strategies, the therapeutic index of isorhamnetin can be improved in the future, as isorhamnetin will mainly exert its effects on the targeted cells, avoiding unnecessary exposures and preventing undesired effects.

### 6.2. Advantages of Isorhamnetin-Based Nanoformulation in Anticancer Applications

**Figure 12:**
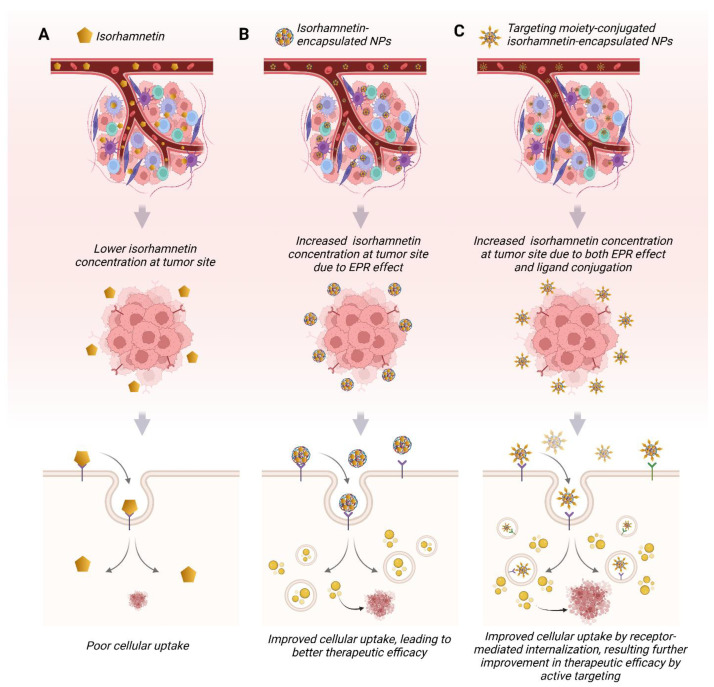
Benefits of utilizing nanocarriers for isorhamnetin drug delivery in cancer therapy. (**A**) Isorhamnetin has low systemic availability due to poor water solubility, inadequate absorption, low stability, rapid metabolism, and quick excretion, which diminishes the active drug concentration at tumor sites. The uptake of free isorhamnetin by the tumor cells is also limited, leading to low therapeutic outcomes. (**B**) Isorhamnetin-loaded, nonfunctionalized nanocarriers improve systemic availability and facilitate higher drug delivery to the tumor microenvironment through the enhanced permeability and retention (EPR) effect, as well as improved cellular penetration, resulting in better therapeutic effects compared to free isorhamnetin. (**C**) In contrast, ligand-functionalized or surface-engineered nanocarriers achieve greater therapeutic benefits than nonfunctionalized versions owing to their effective entry in more significant quantities into the tumor microenvironment via the EPR effect and targeted delivery to tumor cells, thereby enhancing their therapeutic effectiveness while reducing non-specific interactions toxicity [307,309,352,409,410,411].

Nanoformulation provides many benefits in drug delivery, especially in improving the therapeutic effectiveness of bioactive compounds such as flavonoids [392,393,394,395]. By encapsulating drugs within nanocarriers, nanoformulation improves solubility, stability, and bioavailability, addressing key challenges associated with conventional methods [394,396]. The small size and high surface area of NPs enable them to exploit the enhanced permeability and retention (EPR) effect [397,398,399,400,401], easing targeted accumulation in tumors while diminishing systemic toxicity [402,403]. Furthermore, nanoformulation permits controlled and sustained release of the isorhamnetin, prolonging its therapeutic action [404,405]. Functionalizing nanocarriers with targeting ligands further improves specificity, allowing receptor-mediated uptake by cancer cells and improving cellular internalization [406,407,408]. Figure 12 demonstrates the improvement in isorhamnetin’s delivery and therapeutic effectiveness, utilizing nanocarriers and targeted delivery methods. It also represents various stages of optimizing drug delivery, showcasing how nanotechnology and active targeting enhance drug concentration and cellular absorption at the tumor site.

Figure 12A shows when isorhamnetin is administered in its free form without any delivery system. The drug (isorhamnetin) shows limited buildup at the tumor. The low levels of isorhamnetin in the TME result in insufficient cellular absorption of isorhamnetin and reduced therapeutic effectiveness [412]. Figure 12B, isorhamnetin is encapsulated within nanocarriers, significantly improving its delivery to the tumor site. The NPs exploit the enhanced permeability and retention (EPR) effect, a phenomenon where the leaky vasculature and impaired lymphatic drainage in tumors allow NPs to accumulate preferentially in the tumor tissue [413,414]. Figure 12C, where isorhamnetin-encapsulated NPs are further functionalized with targeting ligands (e.g., antibodies, peptides, or small molecules) that specifically bind to receptors overexpressed on cancer cells. This active targeting approach combines the benefits of the EPR effect (passive targeting) with receptor-mediated endocytosis (active targeting) [415]. As a result, this strategy achieves the highest therapeutic efficacy by ensuring precise delivery of isorhamnetin to cancer cells while minimizing off-target effects.

**Figure 13:**
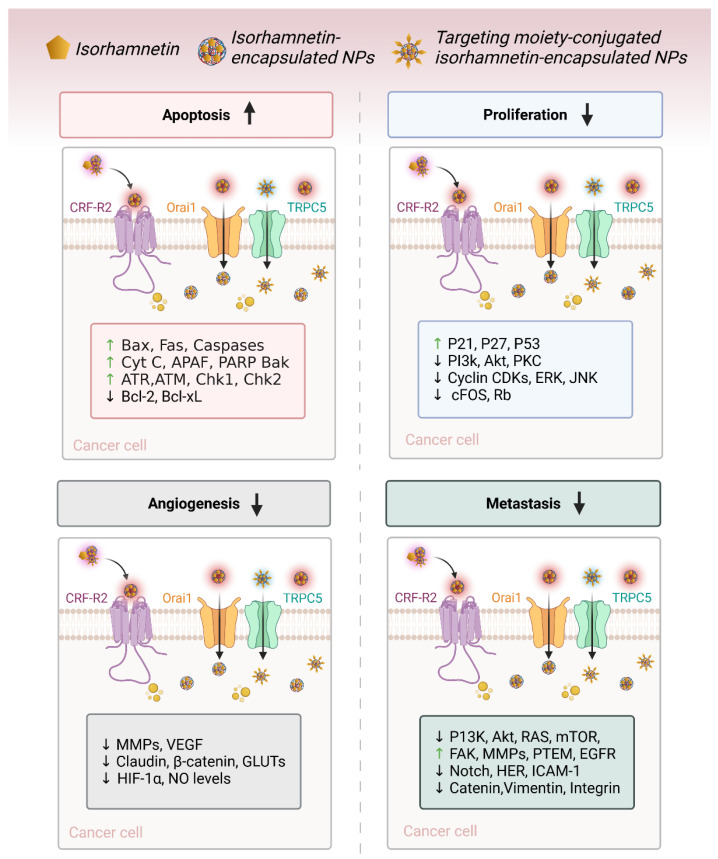
Mechanisms of flavonoid-loaded nanocarriers targeting cancer signaling pathways. Nanocarriers enhance the targeting delivery of flavonoids to specific sites via passive and active mechanisms. Passive targeting utilizes the EPR effect, while active targeting engages receptor-mediated endocytosis. The uptake of isorhamnetin by cancer cells interferes with crucial signaling pathways related to cell proliferation, angiogenesis, metastasis, and apoptosis, thus producing anticancer effects [62,96,144,166,229,269,275,276,277]. The arrows ‘↑’ and ‘↓’ represent activation and suppression, respectively.

Figure 13 illustrates how isorhamnetin, administered through nanocarriers with added targeting moieties, interferes with essential cancer mechanisms—such as proliferation, angiogenesis, and metastasis—by modulating critical molecular pathways. This innovative drug delivery method greatly enhances the therapeutic efficacy of isorhamnetin, presenting a promising approach for targeted cancer treatment.

### 6.3. Challenges

The use of progressive drug delivery systems for isorhamnetin presents numerous challenges that need to be addressed for ideal therapeutic outcomes [350]. One main challenge is warranting the stability of isorhamnetin during the preparation process and the delivery system. Like several other bioactive compounds, Isorhamnetin might experience degradation owing to factors such as heat and pH variations, which can reduce its effectiveness [416,417,418]. Protective encapsulation or coatings are required to overcome such issues, which can add complexity and cost to the formulation process. Another significant challenge is the development of effective and targeted delivery systems. While NPs, microparticles, and other delivery systems can improve the bioavailability and stability of isorhamnetin, they need to be designed carefully to prevent problems such as toxicity, immune responses, or unwanted distribution in non-target organs or tissues [419,420,421]. Achieving accurate targeting and controlled release is hard [422,423], mainly when dealing with complex biological environments that can influence drug release rates or the behavior of the delivery system [424,425,426].

In addition, for methods like transdermal patches, microneedle patches, and drug-loaded contact lenses, warranting acceptable penetration and continuous release of bioactive compounds (isorhamnetin) is a critical challenge [427,428]. The skin may have limited penetrability, demanding further exploration to increase drug absorption and ensure uniform drug release with passing time. Likewise, injectable devices and controlled-release implants must account for factors like local tissue irritation or the capability of the body to absorb the drug appropriately. Lastly, regulatory and manufacturing hurdles pose further challenges. Most of these advanced drug delivery methods necessitate complex manufacturing procedures involving precision engineering and quality control measures, which can increase production costs and time. Moreover, extensive preclinical and clinical testing is required to warrant safety and efficacy. These factors must be carefully considered in future research and developing effective isorhamnetin delivery methods. The advantages and possible challenges of using the advanced delivery system for isorhamnetin are summarized in Table 3.

## 7. Future Perspectives

The therapeutic potential of isorhamnetin for anticancer application has gained significant interest in the past two decades due to its properties, such as antioxidant, anti-inflammatory, and epigenetic modulations [62,89,175]. As our understanding of isorhamnetin’s mechanisms of action evolves, new methodologies and research directions, offering innovative and exciting opportunities for its incorporation into clinical studies and accurate application. These advances could meaningfully improve its utility as an anticancer agent and provide more precise, personalized treatment methods. Here, we discussed some of the future perspectives of isorhamnetin in cancer research and treatment.

### 7.1. Role of Emerging Technologies in Isorhamnetin Research

Emerging technologies in science hold the potential for progressing isorhamnetin-related research and improving its clinical applications. One such area is using high-throughput screening platforms to recognize new isorhamnetin derivatives with better anticancer efficacy and bioavailability [429,430,431]. High-throughput screening helps identify compounds with a similar property to isorhamnetin but with improved pharmacokinetic capabilities for real applications [432,433], such as improved solubility, stability, and higher penetration into the tissues. This can help to accelerate the development of more effective and selective isorhamnetin-based therapies.

CRISPR-Cas9 gene editing technology can also study the molecular mechanisms underlying isorhamnetin’s effects [434]. By selectively knocking out particular genes from cells, researchers can gain deeper insights into the pathways and specific targets through which isorhamnetin exerts its protective (in normal cells) and anticancer effects. This methodology might also assist in identifying genetic variations that may affect distinct responses to isorhamnetin, paving the way for personalized therapies [435].

Furthermore, advancements in nanotechnologies, including the development of further sophisticated drug-delivery systems/methods [436], might also play a crucial role in enhancing the clinical efficacy and real implications of isorhamnetin. NPs and other nanocarriers can improve the solubility, stability, and tumor-targeting abilities of isorhamnetin, ultimately enhancing its bioavailability while reducing the risk of off-target toxicity [437,438,439]. Research into stimuli-responsive nanocarriers—such as those that release the isorhamnetin in response to different body environments, such as pH or temperature changes in the tumor microenvironment—could improve the therapeutic index of isorhamnetin (Figure 11).

### 7.2. Potential for Integration into Precision Oncology

The main goal of precision oncology is to tailor cancer treatment based on the genetic, epigenetic, and molecular mechanisms of specific tumors [440]. As part of this paradigm, isorhamnetin possibly becomes a valuable component of precision cancer therapies [441]. By leveraging genomic and epigenetic profiling, researchers in clinical trials can identify which patients are most likely to benefit from isorhamnetin-based treatment, warranting that the therapy is effective and personalized. The ability of isorhamnetin to modulate multiple cancer-related pathways [66,96,296] makes it a versatile candidate for integration into combination treatments (as explained in Section 5). In precision oncology, isorhamnetin could be combined with other targeted therapies, immunotherapies, chemotherapies, radiation, or NPs to improve therapeutic outcomes.

### 7.3. Limitations and Future Research Directions

Despite the promise of isorhamnetin, several limitations were noted. (i) A substantial amount of in vitro data is presented, but in vivo studies remain relatively limited, restricting a comprehensive understanding. (ii) Toxicological studies on isorhamnetin are insufficient, leaving uncertainties regarding its safety profile. (iii) The mechanisms behind its antihypertensive, antithrombotic, anti-hypoxic, and anti-ultraviolet properties remain unclear. (iv) Few therapeutic targets have been explored, and no modern drug delivery systems have been tested to enhance their bioavailability and targeted therapy. (v) Limited studies have been conducted on combination treatments involving isorhamnetin, such as its synergistic effects with radiation, chemotherapeutic drugs, radiotherapy, NPs, and other natural compounds. (vi) Limited information about the potential binding targets and binding sites. Addressing these gaps will be essential to unlock the therapeutic potential of isorhamnetin fully.

Future research on isorhamnetin should focus on several key areas to unlock its full therapeutic potential. (1) There is a need to increase in vivo studies, particularly those examining its effects on critical signaling pathways, to further elucidate its pharmacological mechanisms. (2) Toxicological studies and investigations into potential drug interactions are vital for establishing its safety profile and progressing its therapeutic importance. (3) Understanding the structure-activity relationship of isorhamnetin and its derivatives, alongside identifying specific pharmacological or binding targets, will also provide deeper insights into its therapeutic applications. (4) Development of advanced drug delivery systems to address the challenges of bioavailability and stability, which have limited its clinical translation. Enhancing these properties will improve its efficacy and tumor-targeting capabilities. (5) Preclinical and clinical studies must confirm its long-term safety, efficacy, and integration into standard cancer treatments. (6) Investigating its role in cancer prevention, genetic susceptibility, and personalized medicine will further broaden its therapeutic scope, offering more effective and safer treatment options. (7) Bioinformatic studies are needed to identify novel proteins and binding sites for targeted treatment. (8) To explore its synergistic potential, Further research is needed on combining isorhamnetin with anticancer drugs, radiotherapy, NPs, and other natural compounds. (9) Artificial intelligence (AI) and machine learning can be used to predict isorhamnetin’s interactions with molecular targets and optimize its structure for enhanced anticancer activity. As these research areas progress, we believe isorhamnetin has the potential to become a cornerstone in modern cancer therapies.

## 8. Conclusions

Isorhamnetin exhibits considerable potential as a multifunctional anticancer agent, with growing evidence supporting its ability to target key hallmarks of cancer biology, including oxidative stress, inflammation, cell cycle regulation, apoptosis, angiogenesis, and metastasis (as detailed in Table 1 and Section 3). Unlike the broader perspective offered by Biswas et al. [20], this review delivers a more focused and updated synthesis of isorhamnetin’s therapeutic activity, highlighting recent advances in combination strategies, immune modulation, and the pharmacological relevance of its glycosidic forms. Mechanistically, isorhamnetin inhibits cancer cell proliferation by modulating cyclins and CDKs, promotes apoptosis through caspase activation and mitochondrial dysfunction, and suppresses metastatic spread by downregulating MMPs, VEGF, and key EMT markers. Additionally, its antioxidant and anti-inflammatory effects help mitigate ROS and pro-inflammatory cytokines, thereby modulating the tumor microenvironment. These combined actions support isorhamnetin’s synergistic potential with chemotherapeutic agents and natural compounds, reinforcing its applicability in multi-targeted combination therapies. Taken together, this review integrates recent mechanistic insights and cancer-type-specific responses to position isorhamnetin as a promising candidate in the evolving landscape of natural-product-based oncology therapeutics.

## Figures and Tables

**Table 3:** The summary of delivery methods, their advantages, and challenges.

Delivery Method	Advantages	Challenges
Nanoparticles	Improved solubility, targeted delivery, enhanced stability	Potential toxicity, complex formulation, high production costs
Cell Targeting	Specific cell delivery, minimal side effects, targeted therapy	Requires identification of specific biomarkers, complex design
Antibody-Drug Conjugate	Precise targeting, reduced off-target effects, higher therapeutic index	Complex design, risk of immune reactions, expensive
Microparticle Depot	Sustained release, controlled release profile, long-term therapy	Slow drug release, potential drug degradation, and formulation challenges
Multiparticulate System	Improved bioavailability, reduced side effects, controlled release	Requires precise formulation, potential for uneven drug distribution
Polymer Film	Controlled release protects the drug from degradation, versatile	May require large doses, limited release rate, potential skin irritation (for transdermal)
pH-Responsive Capsule	Site-specific release protects from stomach acid, enhances absorption	Limited to the gastrointestinal tract, there is potential for incomplete release
Microencapsulation	Enhanced stability, controlled release, protects from degradation	Slow release, complex preparation, limited for rapid onset
Coated Microparticles	Controlled release protects the drug from degradation and enhances stability.	Complex formulation, potential for incomplete release, high production costs
Transdermal Patch	Non-invasive, steady drug release, convenient for chronic conditions	Limited skin permeability, slow absorption, skin irritation
Microneedle Patch	Pain-free targeted drug delivery, easy to use	Limited drug load, potential for skin irritation, expensive
Drug-Loaded Contact Lens	Direct drug delivery to the eye, localized treatment, non-invasive	Limited to ocular diseases, potential irritation, short duration of effect
Controlled Release Implant	Prolonged, consistent release, reduced dosing frequency, localized delivery	Invasive, local tissue irritation, difficult to remove
Swellable Hydrogel	Responsive to fluids, sustained release can be used for wound care	Limited to topical applications, swelling issues, possible irritation
Wound Dressing	Direct drug application to wounds accelerates healing and protects from infection.	Requires proper formulation for sustained release, may need frequent changes
Injectable Device	Rapid onset, precise control of drug dose, targeted delivery	Invasive, potential local irritation, requires medical supervision

## Data Availability

Data are contained within the article.

## Abbreviations

The following abbreviations are used in this manuscript:

ASR	Age-standardized rate
CDK	Cyclin-dependent kinase
EMT	Epithelial–mesenchymal transition
MMP	Matrix metalloproteinase
OSCC	Oral squamous cell carcinoma
VEGF	Vascular endothelial growth factor
ROS	Reactive oxygen species
RNS	Reactive nitrogen species
SOD	Superoxide dismutase
MAPK	Mitogen-activated protein kinase
AMPK/mTOR	AMP-activated protein kinase/Mammalian Target of Rapamycin
pRb	Retinoblastoma Protein
MCL	Myeloid Cell Leukemia
Fas	First apoptosis signal receptor
TRAIL	Tumor Necrosis Factor-Related Apoptosis-Inducing Ligand
FADD	Fas-Associated protein with Death Domain
BID	BH3 Interacting-domain death agonist
APAF	Apoptotic Protease Activating Factor
SMAC	Second Mitochondria-derived Activator of Caspases
ACSL	Acyl-CoA Synthetase Long-Chain Family Member
PTGS	Prostaglandin-Endoperoxide Synthase
DSS	Dextran Sulfate Sodium
VRAP	VEGF Receptor-Associated Protein
ERK	Extracellular Signal-Regulated Kinase
JNK	c-Jun N-terminal Kinase
uPA	Urokinase-type Plasminogen Activator
NSCLC	Non-Small-Cell Lung Cancer
COLA1	Collagen Type I Alpha 1
α-SMA	Alpha-Smooth Muscle Actin
HIF	Hypoxia-Inducible Factor
CTCs	Circulating Tumor Cells
ECM	Extracellular Matrix
TME	Tumor microenvironment
NK	Natural killer
ICD	Immunogenic cell death
DAMP	damage-associated molecular pattern
CAF	Cancer-associated fibroblast
TAM	Tumor-associated macrophage
PFN	Perforin
IFN	Interferon
TNF	Tumor necrosis factor
HO	Heme oxygenase
PAH	Pulmonary arterial hypertension
NPs	Nanoparticles
EPR	Enhanced permeability and retention
GPCR	G-protein coupled receptor
RTK	Receptor tyrosine kinase
